# Role of miRNA in Cardiovascular Diseases in Children—Systematic Review

**DOI:** 10.3390/ijms25020956

**Published:** 2024-01-12

**Authors:** Marta Pasławska, Aleksandra Grodzka, Joanna Peczyńska, Beata Sawicka, Artur Tadeusz Bossowski

**Affiliations:** Department of Pediatrics, Endocrinology, Diabetology with Cardiology Divisions, Medical University of Bialystok, J. Waszyngtona 17, 15-274 Bialystok, Poland; marta.paslawska@sd.umb.edu.pl (M.P.); aleksandra7grodzka@gmail.com (A.G.); jpeczynska@gmail.com (J.P.); beata.sawicka@umb.edu.pl (B.S.)

**Keywords:** microRNA, miRNA, children, pediatric, arrhythmias, myocarditis, cardiomyopathy, congenital heart disease, cardiovascular disease

## Abstract

The number of children suffering from cardiovascular diseases (CVDs) is rising globally. Therefore, there is an urgent need to acquire a better understanding of the genetic factors and molecular mechanisms related to the pathogenesis of CVDs in order to develop new prevention and treatment strategies for the future. MicroRNAs (miRNAs) constitute a class of small non-coding RNA fragments that range from 17 to 25 nucleotides in length and play an essential role in regulating gene expression, controlling an abundance of biological aspects of cell life, such as proliferation, differentiation, and apoptosis, thus affecting immune response, stem cell growth, ageing and haematopoiesis. In recent years, the concept of miRNAs as diagnostic markers allowing discrimination between healthy individuals and those affected by CVDs entered the purview of academic debate. In this review, we aimed to systematise available information regarding miRNAs associated with arrhythmias, cardiomyopathies, myocarditis and congenital heart diseases in children. We focused on the targeted genes and metabolic pathways influenced by those particular miRNAs, and finally, tried to determine the future of miRNAs as novel biomarkers of CVD.

## 1. Introduction

The number of children suffering from cardiovascular diseases (CVDs) such as arrhythmias, cardiomyopathies, myocarditis and congenital heart diseases is rising globally. According to the World Health Organisation, heart disease is not a major cause of death among children and adolescents, however, it is the leading cause of death globally among the entire population [1]. Cardiovascular diseases such as congenital heart defects, cardiomyopathies, arrhythmias and myocarditis may cause critical developmental problems in children, which, in some cases, might lead to premature death. Therefore, there is an urgent need to acquire a better understanding of the genetic factors and molecular mechanisms related to cardiovascular diseases in order to develop new prevention and treatment strategies.

Over the last 30 years, extensive research has revealed the highly complex regulatory networks that control cardiovascular development, underscoring the importance of investigating both genetic and environmental factors in the pathogenesis of CVD, in hopes of identifying reliable biomarkers, specific to particular diseases.

MicroRNAs (miRNAs), discovered in 1993 in *Caenorhabditis elegans*, constitute a class of small non-coding RNA fragments ranging from 17 to 25 nucleotides in length, which are highly conserved across various species [2,3]. miRNAs are able to inhibit protein translation, affect their expression or induce degradation of different mRNAs [4]. Found in serum, plasma, and other body fluids, miRNAs exercise control over an abundance of biological aspects of cell life such as proliferation, differentiation, and apoptosis, thus affecting immune response, stem cell growth, ageing and haematopoiesis [5,6,7].

The most frequently reported miRNAs are transcribed from DNA sequences into primary miRNAs (pri-miRNAs). They are further processed by the enzyme Drosha into precursor miRNAs (pre-miRNAs). Pre-miRNA is subsequently exported from the nucleus to the cytoplasm by Exportin-5 and then transformed into mature miRNAs. In the majority of cases, miRNAs interact with the 3′ UTR or 5′ UTR regions to repress mRNA expression [3,8]. However, miRNAs have also been reported to interact with other regions including coding sequences and gene promoters [9]. Databases such as miRbase or PlasmiR might be useful tools for searching for microRNA gene sequences [4,10].

Only in recent years, has the concept of miRNAs as promising biomarkers for the vast majority of CVDs entered the purview of academic debate: miRNAs may prove to be a major help in discriminating between healthy individuals and those affected by diseases. New, improved methods of miRNA sequencing can also help to achieve this goal [4,11]. Another fascinating perspective arises from studying the relationship between one’s sex and miRNA expression, as it may differ between men and women due to both hormonal and genetic factors [12,13].

In this review, we aimed to search for and describe miRNAs related to the aforementioned CVDs in the paediatric population, focusing on the targeted genes as well as the pathways influenced by those particular miRNAs, and finally, we tried to determine the future of miRNAs as novel biomarkers in CVDs.

## 2. Materials and Methods

**Figure 1:**
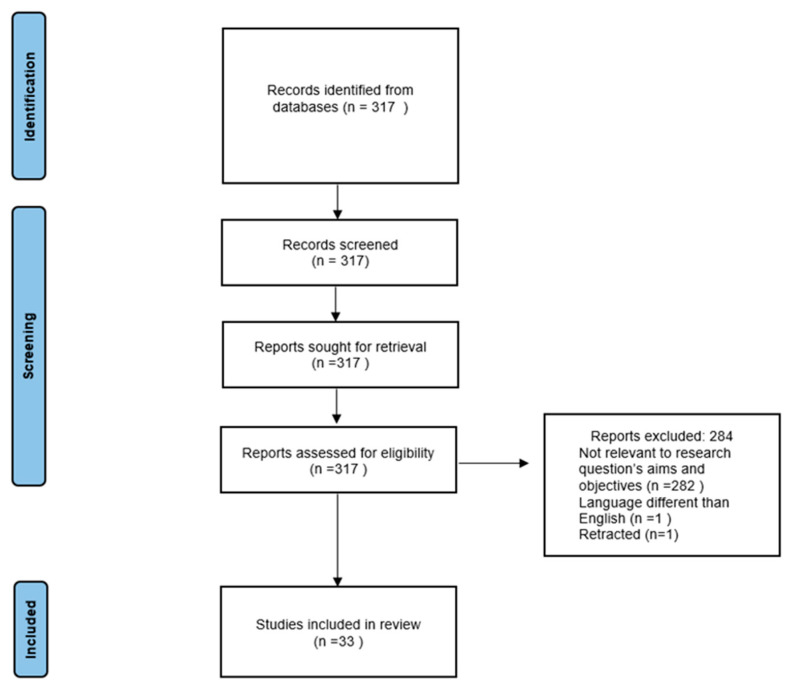
PRISMA 2020 flow diagram of the research process and studies included in the review.

A systematic search was conducted by using the databases Medline (via PubMed), Scopus and Google Scholar. We used the following MeSH terms: “children” or “pediatric” and “miRNA” or “microRNA” or “microRNAs” as well as “arrhythmia”, “cardiomyopathy”, “myocarditis”, “congenital heart diseases”. The search was limited only to original papers and systematic reviews written in English. The review protocol followed the declaration of the Preferred Reporting Items for Systematic Reviews and Meta-Analyses (PRISMA) statement (Figure 1). A total of 317 records were screened from which 33 papers were included in this review, based on selected search criteria. The excluded studies, a total of 284, were not relevant to the research question’s aims and objectives (these were, for instance, ‘in vitro’ studies or research performed on adults), 1 of them was not written in English and 1 paper was retracted (Figure 1).

## 3. Results

### 3.1. Arrhytmias

The number of children requiring diagnosis and treatment for cardiac arrhythmias is increasing [12,14]. In the pediatric population, the most common arrhythmias are supraventricular or ventricular extrasystoles (SVT and VT), which, when advanced, may lead to heart failure (or a patient’s death, if advanced) [12,14]. There is very limited research into miRNAs in arrhythmias in children. To our knowledge, only two original papers dealing with this problem have currently been published.

**Figure 2:**
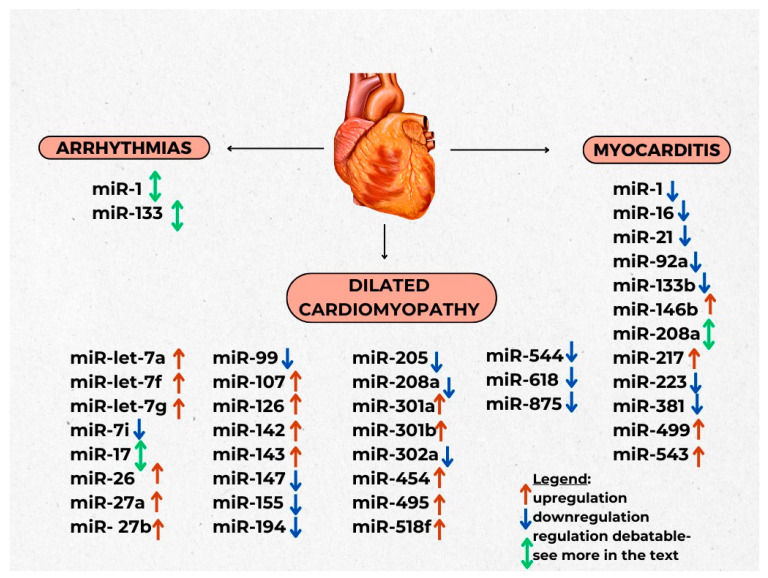
Chosen miRNAs and their regulation in arrhythmias, myocarditis and DCM in children.

Sun et al. (2015) were the first to notice that **miR-1** and **miR-133** may play a significant role in arrhythmias in children (see more in Figure 2 and Supplementary Table S1) [15]. They showed that miR-1 expression levels were lower in patients with arrhythmia compared to controls, whereas there was no noticeable difference between those two groups in terms of miR-133 expression levels. Upregulation of connexin 43, encoded by *GJA1* gene, a protein component of gap junctions involved in the regulation of contraction of the heart muscle, causes inhibition of miR-1, which in turn leads to an increase in myocardial conduction velocity. This phenomenon may explain the pathogenesis of SVT in children. Moreover, researchers proved that miR-1 has high sensitivity and specificity for the evaluation of SVT, whereas miR-133 can be used to evaluate VT [15,16]. On the other hand, Moric-Janiszewska et al. (2021) found that patients with ventricular arrhythmias (Va) such as ventricular extrasystoles, ventricular tachycardia and supraventricular arrhythmias (SVa), supraventricular tachycardia and supraventricular extrasystoles were characterised by different expressions in miR-1, miR-133a and miR-133b. Higher expression levels of miR-1 were observed in SVa patients in comparison to the control group. miR-133a levels were higher in SVa patients than in Va patients and controls whereas miR-133b was statistically lower in SVa and Va patients when compared to controls [12].

**miR-1** plays an important role in regulating the expression of different genes such as syntaxin 6 (*Stx6*) gene [17]. It regulates cardiac conduction by targeting *GJA1* and *KCNJ2*, cardiac automaticity by targeting *HCN2* and *HCN4*, and cardiac repolarisation by targeting *KCNA5*, *KCND2* and *KCNE1*. Furthermore, miR-1 seems to target *SLC8A1*, the gene encoding NXC1 protein. Its upregulation may have an impact on calcium handling and contribute overall to diastolic dysfunction and an increased risk of arrhythmias [18]. In mice, miR-1 was proved to induce atrioventricular block due to inhibiting potassium channel (Kir2.1) expression [19,20].

**miR-133** plays an important role in promoting the differentiation of fibroblasts into cardiomyocyte-like cells [21]. It also targets genes encoding potassium channels (*KCNH2* and *KCNQ1*) and controls cardiac repolarisation. Repressing HERG K+ channel gene KCNH2 in mice with diabetes mellitus led to QT prolongation [22]. However, in humans, miR-133a-1 and miR-133a-2 as well as miR-1-1 and miR-1-2 sequence variants were not causing LQTS [23]. Moreover, **miR-133a/b** downregulation in mice increased adrenergic, Wnt/calcium, and FGFR1 signalling. This might lead to pathological remodelling of cardiomyocytes [24].

### 3.2. Myocarditis

Myocarditis is a severe inflammatory disease of the myocardium, frequently occurring in children and adolescents, often caused by infections with various bacteria, viruses, rickettsiae, fungi and parasites. The most common aetiological agents in the youngest patients are viruses, especially Coxsackie group B (CVB), parvovirus B19, influenza virus or rubella virus (viral cardiomyositis, VCM). Myocarditis may also develop during the course of autoimmune diseases, such as systemic lupus erythematosus, connective tissue diseases or sarcoidosis [25].

A study conducted by Goldberg et al. (2018) shows that, in children, levels of cardiac-associated miRNA such as **miR-208a**, **miR-208b**, **miR-499**, and **miR-21** present certain upward or downward dynamics depending on the phase of enteroviral, adenoviral or parvoviral B19 myocarditis [25]. The level of miR-208a, a molecule that is expressed by cardiomyocytes and released upon myocardial damage, was high during the acute phase and significantly decreased during both the subacute phase and the resolution/chronic phase [26,27]. Levels of **miR-208b** did not change significantly during the subacute and resolution/chronic phases. On the contrary, the level of **miR-499** presented an upward trend during those phases. Nevertheless, those changes did not reach a level of statistical significance [25]. It is worth mentioning that highly elevated levels of **miR-208b** and **miR-499** were observed in adults with viral myocarditis as well [28].

According to Goldberg et al., the level of **miR-21**, which is not only a cardiac-associated but also an inflammatory-related molecule, did not change during the subacute phase, although it decreased significantly during the resolution/chronic phase [25]. On the one hand, Yang et al. (2018) observed that miR-21 deficiency promoted inflammatory cytokine production and worsened cardiac function whereas miR-21 overexpression worked in a reverse manner in the myocardial infarction model [29]. On the other hand, Li et al. (2022) and Gong et al. (2023) argued that miR-21 downregulation protected myocardial cells against lipopolysaccharide-induced apoptosis and inflammation [30,31]. Moreover, inhibition of miR-21 may play a protective role against sepsis-induced cardiac dysfunction [30,31]. Additionally, Goldberg et al. presented correlations between cardiac-associated and inflammatory-associated miRNAs [25]. The significantly downregulated levels of cardiac-associated miR-208a during the subacute phase strongly correlated with the significantly downregulated levels of cardiac- and inflammatory-associated miR-21 during the chronic/resolution phase. This result indicates a link between cardiac damage and immune and inflammatory reactions. Simultaneously, there was no significant relation between the plasma levels of inflammatory miRNAs and the circulating numbers of leukocytes or CRP levels. Researchers also suggested that chosen miRNAs may regulate pathways involved in the immune response [25]. This study shows the potential of using miR-208a as a diagnostic marker of cardiac damage and proves that miR-208b may be used as a prognostic marker for left ventricular function recovery in children with myocarditis.

According to Zhang et al. (2018), the level of **miR-381** in serum and myocardial tissue was lower in both children and mice with viral myocarditis, when compared to the control group of young people who had recovered from viral myocarditis and healthy animal models, respectively. The study also suggests that miR-381 can bind both human and mouse cyclooxygenase (COX-2) mRNA to subsequently regulate their expression [32]. miR-381 was described to have a protective effect on endothelial cells, operating against inflammation in coronary artery disease. In septic adult patients, miR-381 restored the inflammatory response and myocardial dysfunction [32,33,34,35]. Taking all this into consideration, it seems that miR-381 plays an important role in reducing inflammation in cardiac muscle [32,33,34,35].

Levels of **miR-217** and **miR-543** were increased in Chinese children as well as in mice models with viral myocarditis caused by Coxsackievirus B in comparison to healthy individuals. Moreover, Xia et al. (2020) revealed a correlation between serum levels of miR-217, miR-543 and sirtuin 1 (SIRT1), an NAD+-dependent deacetylase associated with the regulation of oxidative stress and inflammatory response. The mRNA level of SIRT1 in the blood samples from children with viral myocarditis was significantly lower when compared to healthy volunteers. In addition, *SIRT1* was found to be a potential target for both miR-217 and miR-543. Researchers argue that miR-217 and miR-543 inhibition may attenuate viral myocarditis by impending the apoptosis of cardiomyocytes and preventing the inflammatory response and oxidative stress by targeting *SIRT1* [36]. This suggests that miR-217 and miR-543 may be potential novel therapeutic targets for the treatment of viral myocarditis in children [36]. What is more, miR-217, as well as miR-543, may also exhibit proangiogenic properties [37].

Other miRNA molecules that may play a significant role in myocarditis in children are **miR-1** and **miR-146b**. The expression of miR-1 in the serum of children with viral myocarditis caused by Coxsackievirus B, respiratory syncytial virus (RSV), parvovirus B19, human herpes virus (HHV) or other viruses was significantly decreased compared to the control group, whereas the serum level of miR-146b was increased substantially in the VCM group. Moreover, serum levels of miR-1 were negatively correlated with left ventricular fractional shortening (FS) and left ventricular ejection fraction (EF), whereas levels of miR-146b were correlated inversely [38]. Furthermore, miR-1 decreased cardiomyocyte apoptosis by mediating the expression of apoptosis-related genes [39]. miR-146b is seen as an inflammation-related miRNA. According to Liu et al. (2013), **miR-146b** is also highly expressed in mice with Coxackievirus B myocarditis. Its inhibition reduced inflammatory lesions and suppressed Th-17 differentiation. The researchers prognose that inhibiting miR-146b may lead to a reduction in the severity of myocarditis [40]. It represses endothelial activation by inhibiting pro-inflammatory pathways, protects cardiomyocytes from injury during ischemia and can be downregulated by hypoxia [41,42,43]. Taking everything into account, this may explain the reason for elevated values of miR-146b in children with myocarditis. Both miR-1 and miR-146b may be used as diagnosis biomarkers for this condition in the future.

Zhang et al. (2017) described the reduced expression of **miR-133b** in cardiomyocytes infected with the Coxackie B virus and in peripheral blood from children with viral myocarditis. It negatively correlated with myocardial injuries. *Rab27b,* which promotes injuries of cardiomyocytes induced by CVB3 infection and facilitates the synthesis and release of cytokines TNF-α and IL-6, was found to be a target for miR-133b. Hence, miR-133b alleviates CVB3 infection-induced myocardial injuries [44].

In children with rheumatic carditis, expression levels of **miR-16-5p**, **miR-92a-3p** and **miR-223-3p** were significantly downregulated. Lower levels of miR-16 may play a preventive role against inflammation [45]. A decrease in miR-92a may play a protective role against ischemia and tissue necrosis as shown in animal studies [46]. Another interesting point is that miR-223 not only regulates immune cell functions by reducing inflammation but may also protect against heart chamber dilatation [47,48,49,50].

### 3.3. Cardiomyopathies

Cardiomyopathies are rare, heterogenous disorders characterised by the presence of structural and functional alterations of the heart muscle with the simultaneous absence of other factors or systemic diseases, which could contribute to dysfunction of the myocardium, such as abnormal loading conditions (e.g., hypertension) or ischemia (e.g., coronary artery disease). They may have a genetic basis (e.g., mutations in genes encoding sarcomere proteins, desmosomes) or a non-genetic basis (e.g., myocarditis, drugs, alcohol, tachyarrhythmias, endocrine diseases). Concerning their pathogenesis, they may be divided further into dilated cardiomyopathy (DCM), hypertrophic cardiomyopathy (HCM), restrictive cardiomyopathy (RCM), arrhythmogenic right ventricular cardiomyopathy (ARVC) and left ventricular non-compaction (LVNC) [13,48,51].

miRNA associated with the occurrence of DCM may influence pathways responsible for mitochondrial function, hypertrophy, inflammatory response and stem cell differentiation. Hailu et. al. (2022) distinguished 393 miRNAs that were differentially expressed in a significant manner between healthy pediatric patients versus DCM. **miR-301a-3p**, **miR-301b-3p**, **miR-495-3p**, **miR-107** were upregulated, whereas **miR-17-5p**, **miR-208a-5p** were downregulated. Moreover, miR-495-3p, miR-17-5p, miR-107 and miR-181c-5p exhibited sex-specific differences in expression [13]. miR-301a promotes embryonic stem cell differentiation to cardiomyocytes and regulates Cofilin-2 (*Cfl2*) which may impact DCM development [52,53]. miR-495 targets *PTEN* and, while inhibited, attenuates the pathological hypertrophy of cardiomyocytes [54,55].

In direct opposition to Hailu et al., Xu et al. (2021) reported that miR-17-5p was upregulated in pathological hypertrophy. According to them, miR-17-5p targets mitofusin 2 (*MFN2*) to activate the PI3K/AKT/mTOR axis, thus reducing autophagy and promoting pathological cardiac hypertrophy [56]. miR-208a targets *Thrap1* and myostatin which are important negative regulators of muscle growth and hypertrophy [57,58]. The role of miR-107 and miR-181 in cardiac hypertrophy is not well-known yet.

In analogy to Hailu et al., Coşkun et al. (2016) isolated 379 types of miRNA in children with DCM. Expressions of two miRNAs (**miR-454**, **miR-518f**) were found to be higher in children with DCM when compared to the control group [59]. The level of miR-454 was also significantly higher in Egyptian children with DCM and in patients with familial DCM caused by Lamin A/C (*LMNA*) gene mutations [60,61]. It is suggested that this miRNA targets insertion/deletion gene polymorphisms of angiotensin-converting enzyme (*ACE*), which consequently links it to the pathogenesis of DCM [62]. miR-454 was also downregulated in diastolic dysfunction of the heart [63]. **miR-518f** targets zinc finger and BTB domain-containing protein 17 (*ZBTB17*) and plays a role in the progression to DCM [64].

Furthermore, Coşkun et al. showed that expression levels of **miR-618**, **miR-875-3p**, **miR-205**, **miR-194**, **miR-302a**, **miR-147**, **miR-544**, **miR-99b**, **miR-155**, **miR-218** were significantly lower in the DCM group [59]. **miR-875-3p** was also significantly lower in Egyptian children with DCM [60]. **miR-875-3p** targets the myopalladin gene (*MYPN*) and **miR-618** targets mutations on tropomyosin alpha (*TPM1*). Regulation of expression of these genes may lead to DCM development [65,66].

Unlike Coşkun et al., Fayez et al. (2022) reported that the miR-194 level was significantly higher in the child population [60]. **miR-194** targets heparin-binding EGF-like growth factor (*HBEGF*) gene which impairs the phosphorylation of ERBB2/B4 tyrosine kinase receptors and in turn leads to severe DCM [67]. **miR-205** targets myocardial zonula adherens protein (*MYZAP*) gene, the knockdown of which is related to DCM [68]. Moreover, miR-205 was related to angiogenesis promotion and cardiomyocyte apoptosis inhibition [69]. **miR-302a** is not only reported to be expressed during cell proliferation, but it also targets the cardiac-specific protein-leucine-rich repeat containing 10 (*Lrrc10*), the knockout of which in mice caused prenatal systolic dysfunction and was responsible for DCM development in postnatal life [70]. Lower levels of **miR-147** led to an increase in inflammation in the myocardium. Furthermore, miR-147 targets and inhibits hyperpolarisation-activated cyclic nucleotide-gated potassium channel 4 (*HCN4*) gene expression, which when upregulated, is supposed to be responsible for causing heart failure and ischemic cardiomyopathy. Therefore, downregulation of miR-147 may result in a deterioration in heart function in DCM patients [71]. Mutations in ankyrin repeat domain 1 (*ANKRD1*) gene, which is targeted by **miR-544**, were found to be related to DCM [72].

In opposition to Coşkun et al., Ramasamy et al. (2018) reported that **miR-99** was significantly upregulated in hypertrophied cardiomyocytes. Furthermore, they showed that the overexpression of miR-99 diverts the physiological hypertrophy to pathological hypertrophy by regulating Akt-1 pathway [73]. miR-99b was reported to control cardiomyogenesis and target genes responsible for regulating epithelial cell proliferation and migration [74,75].

According to Satoh et al. (2011), **miR-155** levels are decreased in tissues obtained from adult DCM patients in comparison to healthy individuals, which supports the findings of Coşkun et al. [76]. However, comparing two groups of children with DCM, one comprising the patients who were qualified for the transplant or died and the other comprising the patients who recovered, Miyamoto et al. (2015) found that miR-155 was significantly upregulated in the less promising group [77]. This miRNA is expressed commonly in activated T/B cells and monocytes/macrophages and targets Jumonji, an AT-rich interactive domain 2 (*JARID2*) gene [78,79]. Therefore, it may have an important impact on the immune system in DCM formation.

**miR-218** regulates RE1-silencing transcription factor (*REST*) and influences cardiomyocyte hypertrophy development if suppressed [80]. Moreover, it targets nexilin F actin- binding protein (*NEXN*)- mutations of this protein destabilise cardiac Z-disks and lead to DCM. It also plays an important role in angiogenesis [81,82].

Miyamoto et al. compared two groups of children with DCM, one consisting of patients that were qualified for transplant or died and the other of patients that recovered, as mentioned previously. **miR-636** was found to be significantly upregulated and **miR-646** and **miR-639** were downregulated in the less promising group [77]. It is worth mentioning that so far miR-636, miR-639 and miR-646 have been mainly described as cancer-related miRNAs. Among others, they were reported to be associated with colon, gastric, ovarian, cervical, breast, endometrial, liver and thyroid cancers [83,84,85,86,87,88,89].

According to Jiao et al. (2018), eight miRNAs were significantly upregulated in DCM pediatric patients, that is, **let-7f-5p**, **let-7g-5p**, **miR-142-5p**, **miR-126-3p**, **miR 143-3p**, **miR-26a-5p**, **miR-27a-3p**, and **miR-27b-3p**. From these, four have been referred to as a useful tool in childhood DCM detection and diagnosis (miR-142-5p, miR-143-3p, miR-27b-3p, and miR-126-3p) [90]. The **miR-142** level was also increased in patients with familial DCM caused by Lamin A/C (*LMNA*) gene mutations [61]. In opposition to Toro et al. (2018), Sharma et al. (2012) reported that miR-142 was downregulated in cardiac hypertrophy which led to the regulation of cytokine signaling in response to haemodynamic stress and improved cardiac functioning [91]. Moreover miR-142 was found to protect mitochondrial function and inhibit the expression of *SH2B1* gene which directly leads to alleviation of cardiac hypertrophy [92]. **miR-143** impacts cardiovascular development by targeting extracellular signal-regulated kinase 5 (*ERK5*) and repressing adducin3 (*add3*). It was upregulated in hypertrophy models and attenuated inflammatory responses, already induced by myocardial hypertrophy [93,94,95]. Overexpression of **miR-27** leads to selective downregulation of *Mstn* gene coding myostatin and *Mdfi* (MyoD family inhibitor) in atrial cardiomyocytes. In reverse, miR-27 overexpression leads to myocardin (*Myocd*) gene upregulation in atrial cardiomyocytes [96]. Interestingly, specific sequences of the miR-23a–miR-27a–miR-24-2 cluster might respond to angiotensin and norepinephrine-driven pro-hypertrophic signaling pathways. This might explain the overexpression of miR-27 in cardiac hypertrophy [97]. Levels of **miR-126-3p** and **miR-let-7g-5p** were significantly increased in the childhood DCM patients with heart failure, compared to non-heart failure childhood DCM patients. What is more, there was a negative correlation between **miR-126-3p and miR-let-7g-5p** expression and ejection fraction. Circulating miRNAs may not only be associated with the progression of heart failure in childhood DCM patients but may also be used as a biomarker of cardiac dysfunction prediction in patients with cardiac hypertrophy [90].

**miR**-**let-7a** was increased in patients with familial DCM caused by Lamin A/C (*LMNA*) gene mutations and seems to possess anti-hypertrophic properties by targeting calmodulin genes [61,98].

Satoh et al. (2011) associates the decrease in **miR-7i** with poor clinical outcomes in patients with DCM [76].

**miR-26a** was found to be significantly increased in Takotsubo cardiomyopathy patients compared with its levels in healthy adults [99].

miR-21, miR-29a, miR-30d and miR-133a upregulation may indicate the presence of myocardial fibrosis in LVNC in adults [51]. Global longitudinal strain (GLS) is useful for assessing the degree of myocardial fibrosis [100]. However, to our knowledge there are no data available about miRNA levels in children with LVNC or any correlation between those molecules and global longitudinal strain (GLS) in children which presents an opportunity for further research in this field.

### 3.4. Congenital Heart Diseases

Congenital heart diseases (CHD) are common causes of morbidity and mortality in the pediatric population. They are becoming more and more common in children worldwide, with an estimated incidence of five to eight per thousand live births. Fortunately, treatment methods have dramatically improved as of late, although the molecular causes of these diseases remain unknown [101,102]. CHD might be divided into cyanotic and acyanotic CHD. Acyanotic heart diseases such as septal defects atrial (ASD), ventricular (VSD) or atrioventricular (AVSD), patent ductus arteriosus (PDA) are the most frequent CHD [102]. Cyanotic congenital heart diseases such as tetralogy of Fallot (ToF) or transposition of great arteries (TGA) are less common dangerous conditions accompanied by chronic hypoxia [103].

**Figure 3:**
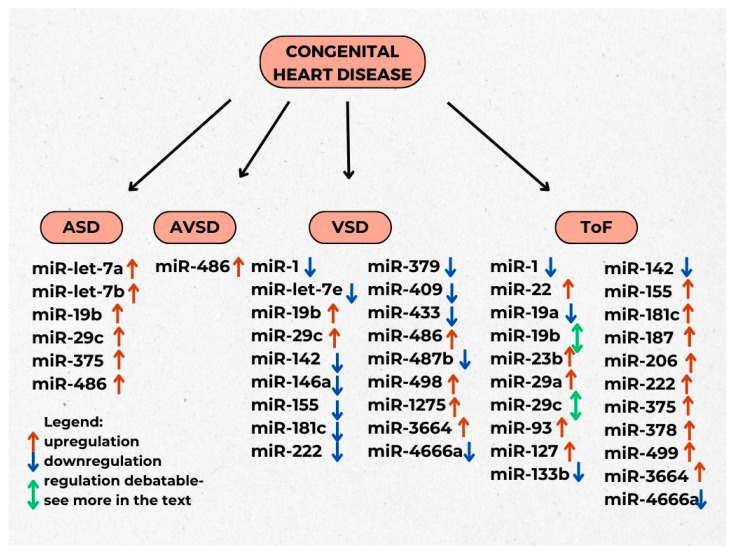
Chosen miRNAs and their regulation in congenital heart diseases in children.

miRNA expression differs in patients with septal defects. On the one hand, Song et al. (2018) have shown that **miR-let-7a** and **miR-let-7b** were specifically related to ASD, but not to other subtypes of septal defects in children (see more in Figure 3). On the other hand, the **miR-486** level was significantly higher in all ASD, VSD and AVSD groups. Surprisingly, parents of CHD children, especially mothers, also had a higher level of circulating miR-let-7a, miR-let-7b and miR-486 compared to parents of healthy children. Those findings suggest that specific types of miRNAs might be associated with specific types of CHD [104]. As mentioned previously, **miR-let-7** possesses anti-hypertrophic properties, whereas miR-486 is modulated by the stretching of cardiac muscle and increases left ventricle growth. Furthermore, miR-486 may regulate cardiomyocyte apoptosis [105,106,107].

Li et al. (2014) reported that in patients with ventricular septal defect (VSD), expression of **miR-498** is upregulated whereas expression of **miR-let-7e-5p**, **miR-155-5p**, **miR-222-3p**, **miR-379-5p**, **miR-409-3p**, **miR-433**, and **miR-487b** is downregulated. miR-let-7e-5p, miR-222-3p and miR-433 were found to target genes related to cardiac development such as *NOTCH1*, *HAND1*, *ZFPM2*, and *GATA3* [108]. **miR-155** not only plays a role in DCM development as previously explained but also regulates *MEF2A*, the deficiency of which in mice caused dilation of the right ventricle, myofibrillar fragmentation, mitochondrial disorganisation and activation of a foetal cardiac gene program and death as a consequence [109]. Other miRNAs associated with VSD are **miR-1-1** and **miR-181c**. miR-1-1, which was downregulated, targeted not only *GJA1* but also *SOX9* genes, whereas miR-181c (upregulated) targeted *BMPR2*. *SOX9* and *BMPR2* are involved in the formation of valves and septa of the heart [16].

Furthermore, Chen et al. reported 78 miRNAs that were differentially expressed in blood samples and lung tissues of pediatric patients with VSD with pulmonary arterial hypertension (PAH). They reported that **miR-19a** may be a novel biomarker for the diagnosis of PAH. Moreover, miR-130a presented proangiogenic properties by targeting, among other genes, *GAX* and *HOXA5* genes. Therefore, these miRNAs may play an important role not only in the regulation of cardiac development but also in angiogenesis and may become promising molecular targets for reversing the remodelling seen in PAH [110].

According to Li et al. (2019), **miR-204** was negatively correlated with the degree of pulmonary hypertension in children with CHD featuring left-to-right shunts such as VSD, ASD or PDA. Moreover, they observed a decreasing trend in miR-204 serum levels after CHD surgery [111].

miRNAs may also be used as prenatal biomarkers of CHD due to their ability to pass through the placental barrier and their stability in maternal circulation. Jin et al. (2021) described 77 differently expressed miRNAs in blood samples obtained from pregnant women with VSD-affected foetuses. The most important one, **miR-146a5**, which targets *PMAIP1*, *NUMB*, *ERBB4*, *IRAK1* and *CCL5* genes that are related to the development and morphogenesis of the heart muscle, was found to effectively distinguish cases of foetal VSD from controls. Therefore, it may potentially be used as a biomarker for prenatal detection of this condition [112].

Zhu et al. (2013) determined that **miR-19b**, **miR-22**, **miR-29c** and **miR-375** might also be used as a prenatal marker of CHD in a foetus. It seems that **miR-19b** and **miR-29c** upregulation correlates especially with VSD [113]. The miR-29 family suppresses excess collagen expression and it may also promote cardiac hypertrophy and CHD development. **miR-29c-3p**, in particular, regulates *Akt3* gene expression whereas miR-29b inhibits cardiomyocyte proliferation via *NOTCH2* [114]. miR-375 may also disrupt cardiomyocyte differentiation via influencing the Notch pathway [115]. Moreover, Zhu et al. (2013) found that **miR-22** targets genes involved in hypertrophy development and may be specifically upregulated in ToF [113,116].

Gu et al. (2019) identified four pregnancy-related miRNAs (**miR-142-5p**, **miR-1275**, **miR-4666a-3p** and **miR-3664-3p**) that may be used to distinguish foetuses with VSD, ToF, single ventricle (SV) and persistent truncus arteriosus (PTA) from the healthy ones. All of these miRNAs were significantly different in VSD. Three miRNAs (miR-142-5p, miR-4666a-3p and miR-3664-3p) were dysregulated in ToF whereas only two miRNAs (miR-142-5p and miR-3664-3p) showed significantly different expression in both SV and PTA [117]. miR-142 plays an important role in cardiac hypertrophy as mentioned previously. The role of miR-4666a-3p and miR-3664-3p in cardiac diseases is not well-known yet.

You et al. (2020) provided a large analysis of miRNAs in ToF, targeted genes and regulated pathways. The most important ones were **miR-499**, **miR-155**, **miR-23b**, **and miR-222**, **miR-93**, **miR-1275** and **miR-187**. miR-499, miR-155, miR-23b, and miR-222 participate in cardiac development [118]. Moreover, miR- 499 and miR-1275 may play a significant role in cardiac muscle mitochondrial functioning whereas miR-23b via targeting the GATA6/IGF-1 axis may promote congenital heart disease development. **miR-93** suppresses cardiac hypertrophy responses and targets cyclin D1 gene (*CCND1*). Disruption in the miR-93/CCND1 signalling pathway was responsible for the development of ventricular remodelling [119,120]. **miR-187** targets *Itpkc*, *Tbl1xr1*, and *Lrrc59* genes responsible for regulating cardiomyocyte apoptosis and cardiac inflammation [118,121].

**miR-222** was also described by Zhang et al. (2013) who showed 18 miRNAs that were variously expressed in ToF and normal myocardial tissues excised from the RVOT. From those, 16 miRNAs were found to target 97 genes involved in heart development. miR-222 was upregulated in ventricular outflow tract tissues from infants with non-syndromic ToF, causing increased cell proliferation and inhibiting cardiomyogenic differentiation. **miR-424** that targeted two heart development genes (*NF1* and *HAS2*) was found to promote cell proliferation and inhibit migration in primary embryonic mouse cardiomyocytes [122].

On the other hand, Grunert et al. (2019) showed that 111 miRNAs were upregulated in ToF and were most importantly heart and muscle related (**miR-206**, **miR-29a-5p**, **miR-378**, and **miR-127**). On the other hand, 61 miRNAs were downregulated including **miR-1**, **miR-133b** (both related to cardiac hypertrophy as mentioned previously) **miR-19a/b-3p**, and **miR-29c** [123].

Wang et al. (2018) discovered that **miR-1** and **miR-133** might be responsible for the variable expression of small RNA (sRNA) between sexes in ToF [124].

Bittel et al. (2014) described that **miR-421** had the greatest change of expression in the RV tissue from infants with ToF. They proved that there was an inverse correlation between the expression of miR-421 and *SOX4*, a key regulator of the Notch and Wnt pathways [125].

It is also worth mentioning that **miR-34a** in mice increased the risk of CHD occurrence by downregulation of *NOTCH-1*, thus modulating Notch signalling pathway [126].

Cardiac hypertrophy caused by CHD was found to be related to higher expression of **miR-1**, **miR-18b**, **miR-21**, **miR-23b**, **miR-133a**, **miR-195**, and **miR-208b** in heart tissue. According to Sánchez-Gómez et al. (2017), miR-21, -23a and -24 can be considered specific biomarkers for the diagnosis of cardiac hypertrophy in infants with CHD [127]. Expression levels of miR-24 were found to strongly correlate with *GATA-4* and *MEF2c* transcription factors that are linked to the heart’s development, regulating the differentiation of precardiac mesoderm and morphogenesis. miR-1 was strongly associated with cell damage and miR-133a moderated the expression of beta-myosin heavy chains (β-MHC) in children with CH [127]. To elaborate further on research carried out by to Sánchez-Gómez et al., Zloto et al. (2020) found **miR-208a** to be of relevance as a promising biomarker of postoperative complications in pediatric patients with CHD who underwent surgery [128].

**miR-219-5p** was found to be significantly upregulated in cyanotic congenital heart disease but not in acyanotic CHD. It seems that the level of **miR-219-5p** increases gradually in hypoxic conditions in a time-dependent manner. Additionally, miR-219-5p binds directly to liver receptor homolog-1 (*LRH-1*); therefore, its downregulation may inhibit hypoxia-induced cardio-myocyte apoptosis [103].

Moreover, Hu et al. (2020) reported that **miR-184** was downregulated in patients with cyanotic congenital heart disease [103]. Inhibition of **miR-184** caused a decrease in cell viability and an induction of apoptosis under hypoxia. Levels of apoptotic proteins caspase-3 and caspase-9 significantly increased due to miR-184 inhibition. Therefore, inhibiting miR-184 might play a protective role against hypoxia in cardiac muscle [129].

**miR-182** was reported to alleviate CHD development due to suppressing hairy and enhancer of split-1 (*HES1*) [130].

**miR-199a-5p** attenuated endoplasmic reticulum stress in cyanotic CHD which posed a protecting effect on cardiomyocytes as a result [131].

Sucharov et al. (2015) presented the unique miRNA profile of the hypoplastic left heart syndrome (HLHS) population. They observed no significant difference between miRNA expression when comparing HLHS patients with right ventricle (RV) failure to those without RV failure. However, there was a significant change in miRNA (**miR-100**, **miR-99** and **miR-145**) expression level in correlation with the stage of surgery. This data might suggest that the volume unloading of the ventricle has important consequences for gene expression [132]. **miR-100**, which can regulate natriuretic peptide receptor 3 (NPR3), was upregulated and protected mice hearts subjected to pressure overload [133,134]. Moreover miR-100 regulates cardiomyocyte hypoxia-induced apoptosis by suppressing the expression of insulin-like growth factor 1 receptor (*IGF1R*) [135]. **miR-99** was upregulated in pathological cardiac hypertrophy [73]. **miR-145** was found to regulate frataxin (*FXN*) gene, which is an important mitochondrial protein that allows maintaining the function of this organellum. By targeting *FXN* gene, miR-145 may influence the apoptosis and mitochondrial function and regulate the development of CHD [136].

Interestingly, even single-nucleotide polymorphisms in miRNA-machinery genes may impact miRNA processing efficiency or function. Borghini et al. (2021) showed that polymorphisms of *XPO5* gene, responsible for the transport of pre-miRNAs between the nucleus and cytoplasm, may impact CHD development [137].

## 4. Conclusions

There is still little known about miRNAs’ impact on cardiovascular diseases in children. Further studies based on larger study groups have to be performed in order to obtain sufficient data. However, it seems that by targeting various genes, miRNAs might play a major role in the development of the CVDs such as arrhythmias, cardiomyopathies, myocarditis and congenital heart diseases in the paediatric population. Providing future researchers inquire more closely into the role miRNAs play in the pathogenesis of CVDs in children, miRNAs might be popularised as useful novel biomarkers and potentially become an important genetic counselling tool.

## Data Availability

The data used to support the findings of this study are available from the corresponding author.

## Supplementary Materials

The following supporting information can be downloaded at: https://www.mdpi.com/article/10.3390/ijms25020956/s1. Refs. [138,139,140,141,142,143,144,145,146,147,148,149,150,151,152,153,154,155,156,157,158,159,160,161,162,163,164,165] are cited in the Supplementary Materials.

## References

[1] The Top 10 Causes of Death. https://www.Who.Int/News-Room/Fact-Sheets/Detail/The-Top-10-Causes-Of-Death?Fbclid=Iwar2llcq33xsxbhssptiozzuynislhja4oroomf3cqs-Svkzfutpy20x7heo.

[2] Lee R.C., Feinbaum R.L., Ambrost V. (1993). The *C. elegans* Heterochronic Gene Lin-4 Encodes Small RNAs with Antisense Complementarity to Lin-14. Cell.

[3] O’Brien J., Hayder H., Zayed Y., Peng C. (2018). Overview of MicroRNA Biogenesis, Mechanisms of Actions, and Circulation. Front. Endocrinol..

[4] Tastsoglou S., Miliotis M., Kavakiotis I., Alexiou A., Gkotsi E.C., Lambropoulou A., Lygnos V., Kotsira V., Maroulis V., Zisis D. (2021). Plasmir: A Manual Collection of Circulating Micrornas of Prognostic and Diagnostic Value. Cancers.

[5] Vaschetto L.M. (2018). MiRNA Activation Is an Endogenous Gene Expression Pathway. RNA Biol..

[6] Dietrich C., Singh M., Kumar N., Singh S.R. (2018). The Emerging Roles of MicroRNAs in Stem Cell Aging. Advances in Experimental Medicine and Biology.

[7] Xu P., Vernooy S.Y., Guo M., Hay B.A. (2003). The Drosophila MicroRNA Mir-14 Suppresses Cell Death and Is Required for Normal Fat Metabolism. Curr. Biol..

[8] Ha M., Kim V.N. (2014). Regulation of MicroRNA Biogenesis. Nat. Rev. Mol. Cell. Biol..

[9] Broughton J.P., Lovci M.T., Huang J.L., Yeo G.W., Pasquinelli A.E. (2016). Pairing beyond the Seed Supports MicroRNA Targeting Specificity. Mol. Cell.

[10] Jordan-Alejandre E., Campos-Parra A.D., Castro-López D.L., Silva-Cázares M.B. (2023). Potential MiRNA Use as a Biomarker: From Breast Cancer Diagnosis to Metastasis. Cells.

[11] Condrat C.E., Thompson D.C., Barbu M.G., Bugnar O.L., Boboc A., Cretoiu D., Suciu N., Cretoiu S.M., Voinea S.C. (2020). MiRNAs as Biomarkers in Disease: Latest Findings Regarding Their Role in Diagnosis and Prognosis. Cells.

[12] Moric-Janiszewska E., Smolik S., Morka A., Szydłowski L., Kapral M. (2021). Expression Levels of Serum Circulating MicroRNAs in Pediatric Patients with Ventricular and Supraventricular Arrhythmias. Adv. Med. Sci..

[13] Hailu F.T., Karimpour-Fard A., Toni L.S., Bristow M.R., Miyamoto S.D., Stauffer B.L., Sucharov C.C. (2022). Integrated Analysis of MiRNA–MRNA Interaction in Pediatric Dilated Cardiomyopathy. Pediatr. Res..

[14] Jat K.R., Lodha R., Kabra S.K. (2011). Arrhythmias in Children. Indian J. Pediatr..

[15] Sun L., Sun S., Zeng S., Li Y., Pan W., Zhang Z. (2015). Expression of Circulating MicroRNA-1 and MicroRNA-133 in Pediatric Patients with Tachycardia. Mol. Med. Rep..

[16] Li J., Cao Y., Ma X.J., Wang H.J., Zhang J., Luo X., Chen W., Wu Y., Meng Y., Zhang J. (2013). Roles of MiR-1-1 and MiR-181c in Ventricular Septal Defects. Int. J. Cardiol..

[17] Su X., Liang H., Wang H., Chen G., Jiang H., Wu Q., Liu T., Liu Q., Yu T., Gu Y. (2017). Over-Expression of MicroRNA-1 Causes Arrhythmia by Disturbing Intracellular Trafficking System. Sci. Rep..

[18] Luo X., Zhang H., Xiao J., Wang Z. (2010). Cellular Physiology Regulation of Human Cardiac Ion Channel Genes by MicroRNAs: Theoretical Perspective and Pathophysiological Implications. Cell. Physiol. Biochem..

[19] Zhang Y., Sun L., Zhang Y., Liang H., Li X., Cai R., Wang L., Du W., Zhang R., Li J. (2013). Overexpression of MicroRNA-1 Causes Atrioventricular Block in Rodents. Int. J. Biol. Sci..

[20] Wahl C.M., Schmidt C., Hecker M., Ullrich N.D. (2022). Distress-Mediated Remodeling of Cardiac Connexin-43 in a Novel Cell Model for Arrhythmogenic Heart Diseases. Int. J. Mol. Sci..

[21] Xu Y., Yin G., Jia D., Dou J., Liu X., Guo Z. (2022). Fibroblast-Derived Exosomal MiRNA-133 Promotes Cardiomyocyte-Like Differentiation. Acta Histochem..

[22] Xiao J., Luo X., Lin H., Zhang Y., Lu Y., Wang N., Zhang Y., Yang B., Wang Z. (2007). MicroRNA MiR-133 Represses HERG K+ Channel Expression Contributing to QT Prolongation in Diabetic Hearts. J. Biol. Chem..

[23] Hedley P.L., Carlsen A.L., Christiansen K.M., Kanters J.K., Behr E.R., Corfield V.A., Christiansen M. (2014). MicroRNAs in Cardiac Arrhythmia: DNA Sequence Variation of MiR-1 and MiR-133A in Long QT Syndrome. Scand. J. Clin. Lab. Investig..

[24] Cheng W.L., Kao Y.H., Chao T.F., Lin Y.K., Chen S.A., Chen Y.J. (2019). MicroRNA-133 Suppresses ZFHX3-Dependent Atrial Remodelling and Arrhythmia. Acta Physiol..

[25] Goldberg L., Tirosh-Wagner T., Vardi A., Abbas H., Pillar N., Shomron N., Nevo-Caspi Y., Paret G. (2018). Circulating MicroRNAs: A Potential Biomarker for Cardiac Damage, Inflammatory Response, and Left Ventricular Function Recovery in Pediatric Viral Myocarditis. J. Cardiovasc. Transl. Res..

[26] Devaux Y., Vausort M., Goretti E., Nazarov P.V., Azuaje F., Gilson G., Corsten M.F., Schroen B., Lair M.L., Heymans S. (2012). Use of Circulating MicroRNAs to Diagnose Acute Myocardial Infarction. Clin. Chem..

[27] Wang H., Bei Y., Shen S., Huang P., Shi J., Zhang J., Sun Q., Chen Y., Yang Y., Xu T. (2016). MiR-21-3p Controls Sepsis-Associated Cardiac Dysfunction via Regulating SORBS2. J. Mol. Cell. Cardiol..

[28] Corsten M.F., Dennert R., Jochems S., Kuznetsova T., Devaux Y., Hofstra L., Wagner D.R., Staessen J.A., Heymans S., Schroen B. (2010). Circulating MicroRNA-208b and MicroRNA-499 Reflect Myocardial Damage in Cardiovascular Disease. Circ. Cardiovasc. Genet..

[29] Yang L., Wang B., Zhou Q., Wang Y., Liu X., Liu Z., Zhan Z. (2018). MicroRNA-21 Prevents Excessive Inflammation and Cardiac Dysfunction after Myocardial Infarction through Targeting KBTBD7. Cell Death Dis..

[30] Gong M., Tao L., Li X. (2023). MicroRNA-21-3p/Rcan1 Signaling Axis Affects Apoptosis of Cardiomyocytes of Sepsis Rats. Gen. Physiol. Biophys..

[31] Li Y., Sun G., Wang L. (2022). MiR-21 Participates in LPS-Induced Myocardial Injury by Targeting Bcl-2 and CDK6. Inflamm. Res..

[32] Zhang Y., Sun L., Sun H., Yu Z., Liu X., Luo X., Li C., Sun D., Li T. (2018). MicroRNA-381 Protects Myocardial Cell Function in Children and Mice with Viral Myocarditis via Targeting Cyclooxygenase-2 Expression. Exp. Ther. Med..

[33] Liu J., Yang Y., Lu R., Liu Q., Hong S., Zhang Z., Hu G. (2021). MicroRNA-381-3p Signatures as a Diagnostic Marker in Patients with Sepsis and Modulates Sepsis-Steered Cardiac Damage and Inflammation by Binding HMGB1. Bioengineered.

[34] Lu L., Zhang H., Dong W., Peng W., Yang J. (2018). MiR-381 Negatively Regulates Cardiomyocyte Survival by Suppressing Notch Signaling. In Vitro Cell. Dev. Biol. Anim..

[35] Li Y., Huang J., Yan H., Li X., Ding C., Wang Q., Lu Z. (2020). Protective Effect of MicroRNA-381 against Inflammatory Damage of Endothelial Cells during Coronary Heart Disease by Targeting CXCR4. Mol. Med. Rep..

[36] Xia K., Zhang Y., Sun D. (2020). MiR-217 and MiR-543 Downregulation Mitigates Inflammatory Response and Myocardial Injury in Children with Viral Myocarditis by Regulating the SIRT1/AMPK/NF-κB Signaling Pathway. Int. J. Mol. Med..

[37] Staszel T., Zapała B., Polus A., Sadakierska-Chudy A., Kieć-Wilk B., Stępień E., Wybrańska I., Chojnacka M., Dembińska-Kieć A. (2011). Role of MicroRNAs in Endothelial Cell Pathophysiology. Pol. Arch. Med. Wewn..

[38] Wang D., Li T., Cui H., Zhang Y. (2016). Analysis of the Indicating Value of Cardiac Troponin I, Tumor Necrosis Factor-α, Interleukin-18, Mir-1 and Mir-146b for Viral Myocarditis among Children. Cell. Physiol. Biochem..

[39] Li W., Liu M., Zhao C., Chen C., Kong Q., Cai Z., Li D. (2020). MiR-1/133 Attenuates Cardiomyocyte Apoptosis and Electrical Remodeling in Mice with Viral Myocarditis. Cardiol. J..

[40] Liu Y.L., Wu W.F., Xue Y., Gao M., Yan Y., Kong Q., Pang Y., Yang F. (2013). MicroRNA-21 and -146b Are Involved in the Pathogenesis of Murine Viral Myocarditis by Regulating TH-17 Differentiation. Arch. Virol..

[41] Di Y.-F., Li D.-C., Shen Y.-Q., Wang C.-L., Zhang D.-Y., Shang A.-Q., Hu T. (2017). MiR-146b Protects Cardiomyocytes Injury in Myocardial Ischemia/Reperfusion by Targeting Smad4. Am. J. Transl. Res..

[42] Chouvarine P., Legchenko E., Geldner J., Riehle C., Hansmann G. (2019). Hypoxia Drives Cardiac MiRNAs and Inflammation in the Right and Left Ventricle. J. Mol. Med..

[43] Cheng H.S., Sivachandran N., Lau A., Boudreau E., Zhao J.L., Baltimore D., Delgado-Olguin P., Cybulsky M.I., Fish J.E. (2013). MicroRNA-146 Represses Endothelial Activation by Inhibiting pro-Inflammatory Pathways. EMBO Mol. Med..

[44] Zhang Y., Sun L., Sun H., Liu X., Luo X., Li C., Sun D., Li T. (2017). Overexpression of MicroRNA-133b Reduces Myocardial Injuries in Children with Viral Myocarditis by Targeting Rab27B Gene. Cell. Mol. Biol..

[45] Gumus G., Giray D., Bobusoglu O., Tamer L., Karpuz D., Hallioglu O. (2018). MicroRNA Values in Children with Rheumatic Carditis: A Preliminary Study. Rheumatol. Int..

[46] Bonauer A., Carmona G., Iwasaki M., Mione M., Koyanagi M., Fischer A., Burchfield J., Fox H., Doebele C., Ohtani K. (2009). MicroRNA-92a Controls Angiogenesis and Functional Recovery of Ischemic Tissues in Mice. Science.

[47] Pan L., Yan B., Zhang J., Zhao P., Jing Y., Yu J., Hui J., Lu Q. (2022). Mesenchymal Stem Cells-Derived Extracellular Vesicles-Shuttled MicroRNA-223-3p Suppress Lipopolysaccharide-Induced Cardiac Inflammation, Pyroptosis, and Dysfunction. Int. Immunopharmacol..

[48] Stauffer B.L., Russell G., Nunley K., Miyamoto S.D., Sucharov C.C. (2013). MiRNA Expression in Pediatric Failing Human Heart. J. Mol. Cell. Cardiol..

[49] Chen L., Hou X., Zhang M., Zheng Y., Zheng X., Yang Q., Li J., Gu N., Zhang M., Sun Y. (2020). MicroRNA-223-3p Modulates Dendritic Cell Function and Ameliorates Experimental Autoimmune Myocarditis by Targeting the NLRP3 Inflammasome. Mol. Immunol..

[50] Gou W., Zhang Z., Yang C., Li Y. (2018). MiR-223/Pknox1 Axis Protects Mice from CVB3-Induced Viral Myocarditis by Modulating Macrophage Polarization. Exp. Cell Res..

[51] Szemraj J., Masiarek K., Majos A., Szemraj-Rogucka Z.M. (2019). Circulating MicroRNAs as Biomarkers for Myocardial Fibrosis in Patients with Left Ventricular Non-Compaction Cardiomyopathy. Arch. Med. Sci..

[52] Rangrez A.Y., Hoppe P., Kuhn C., Zille E., Frank J., Frey N., Frank D. (2017). MicroRNA MiR-301a Is a Novel Cardiac Regulator of Cofilin-2. PLoS ONE.

[53] Zhen L.-X., Gu Y.-Y., Zhao Q., Zhu H.-F., Lü J.-H., Li S.-J., Xu Z., Li L., Yu Z.-R. (2019). MiR-301a Promotes Embryonic Stem Cell Differentiation to Cardiomyocytes. World J. Stem Cells.

[54] Clark A.L., Maruyama S., Sano S., Accorsi A., Girgenrath M., Walsh K., Naya F.J. (2016). MiR-410 and MiR-495 Are Dynamically Regulated in Diverse Cardiomyopathies and Their Inhibition Attenuates Pathological Hypertrophy. PLoS ONE.

[55] Fu J., Chen Y., Li F. (2018). Attenuation of MicroRNA-495 Derepressed PTEN to Effectively Protect Rat Cardiomyocytes from Hypertrophy. Cardiology.

[56] Xu X., Su Y.-L., Shi J.-Y., Lu Q., Chen C. (2021). MicroRNA-17-5p Promotes Cardiac Hypertrophy by Targeting Mfn2 to Inhibit Autophagy. Cardiovasc. Toxicol..

[57] Callis T.E., Pandya K., Hee Y.S., Tang R.H., Tatsuguchi M., Huang Z.P., Chen J.F., Deng Z., Gunn B., Shumate J. (2009). MicroRNA-208a Is a Regulator of Cardiac Hypertrophy and Conduction in Mice. J. Clin. Investig..

[58] Huang X.-H., Li J.-L., Li X.-Y., Wang S.-X., Jiao Z.-H., Li S.-Q., Liu J., Ding J. (2021). MiR-208a in Cardiac Hypertrophy and Remodeling. Front. Cardiovasc. Med..

[59] Enes Coşkun M., Kervancioǧlu M., Öztuzcu S., Yilmaz Coşkun F., Ergün S., Başpinar O., Kilinç M., Temel L., Coşkun M.Y. (2016). Plasma MicroRNA Profiling of Children with Idiopathic Dilated Cardiomyopathy. Biomarkers.

[60] Fayez A.G., Esmaiel N.N., Salem S.M., Ashaat E.A., El-Saiedi S.A., El Ruby M.O. (2022). MiR-454-3p and MiR-194-5p Targeting Cardiac Sarcolemma Ion Exchange Transcripts Are Potential Noninvasive Diagnostic Biomarkers for Childhood Dilated Cardiomyopathy in Egyptian Patients. Egypt. Heart J..

[61] Toro R., Blasco-Turrión S., Morales-Ponce F.J., Gonzalez P., Martínez-Camblor P., López-Granados A., Brugada R., Campuzano O., Pérez-Serra A., Rosa Longobardo F. (2018). Plasma MicroRNAs as Biomarkers for Lamin A/C-Related Dilated Cardiomyopathy. J. Mol. Med..

[62] Mahjoub S., Mehri S., Bousaada R., Ouarda F., Zaroui A., Zouari B., Mechmeche R., Hammami M., Ben Arab S. (2010). Association of ACE I/D Polymorphism in Tunisian Patients with Dilated Cardiomyopathy. JRAAS-J. Renin-Angiotensin-Aldosterone Syst..

[63] Nair N., Kumar S., Gongora E., Gupta S. (2013). Circulating MiRNA as Novel Markers for Diastolic Dysfunction. Mol. Cell. Biochem..

[64] Li X., Luo R., Mo X., Jiang R., Kong H., Hua W., Wu X. (2013). Polymorphism of ZBTB17 Gene Is Associated with Idiopathic Dilated Cardiomyopathy: A Case Control Study in a Han Chinese Population. Eur. J. Med. Res..

[65] Meyer T., Ruppert V., Ackermann S., Richter A., Perrot A., Sperling S.R., Posch M.G., Maisch B., Pankuweit S. (2013). Novel Mutations in the Sarcomeric Protein Myopalladin in Patients with Dilated Cardiomyopathy. Eur. J. Hum. Genet..

[66] Redwood C., Robinson P. (2013). Alpha-Tropomyosin Mutations in Inherited Cardiomyopathies. J. Muscle Res. Cell Motil..

[67] Friedrichs F., Zugck C., Rauch G.J., Ivandic B., Weichenhan D., Müller-Bardorff M., Meder B., Mokhtari N.E.E., Regitz-Zagrosek V., Hetzer R. (2009). HBEGF, SRA1, and IK: Three Cosegregating Genes as Determinants of Cardiomyopathy. Genome Res..

[68] Seeger T.S., Frank D., Rohr C., Will R., Just S., Grund C., Lyon R., Luedde M., Koegl M., Sheikh F. (2010). Myozap, a Novel Intercalated Disc Protein, Activates Serum Response Factor-Dependent Signaling and Is Required to Maintain Cardiac Function In Vivo. Circ. Res..

[69] Wang T., Li T., Niu X., Hu L., Cheng J., Guo D., Ren H., Zhao R., Ji Z., Liu P. (2023). ADSC-Derived Exosomes Attenuate Myocardial Infarction Injury by Promoting MiR-205-Mediated Cardiac Angiogenesis. Biol. Direct.

[70] Brody M.J., Hacker T.A., Patel J.R., Feng L., Sadoshima J., Tevosian S.G., Balijepalli R.C., Moss R.L., Lee Y. (2012). Ablation of the Cardiac-Specific Gene Leucine-Rich Repeat Containing 10 (Lrrc10) Results in Dilated Cardiomyopathy. PLoS ONE.

[71] Stillitano F., Lonardo G., Zicha S., Varro A., Cerbai E., Mugelli A., Nattel S. (2008). Molecular Basis of Funny Current (If) in Normal and Failing Human Heart. J. Mol. Cell. Cardiol..

[72] Duboscq-Bidot L., Charron P., Ruppert V., Fauchier L., Richter A., Tavazzi L., Arbustini E., Wichter T., Maisch B., Komajda M. (2009). Mutations in the ANKRD1 Gene Encoding CARP Are Responsible for Human Dilated Cardiomyopathy. Eur. Heart J..

[73] Ramasamy S., Velmurugan G., Rekha B., Anusha S., Shanmugha Rajan K., Shanmugarajan S., Ramprasath T., Gopal P., Tomar D., Karthik K.V. (2018). Egr-1 Mediated Cardiac MiR-99 Family Expression Diverges Physiological Hypertrophy from Pathological Hypertrophy. Exp. Cell Res..

[74] Coppola A., Romito A., Borel C., Gehrig C., Gagnebin M., Falconnet E., Izzo A., Altucci L., Banfi S., Antonarakis S.E. (2014). Cardiomyogenesis Is Controlled by the MiR-99a/Let-7c Cluster and Epigenetic Modifications. Stem Cell Res..

[75] Chen D., Chen Z., Jin Y., Dragas D., Zhang L., Adjei B.S., Wang A., Dai Y., Zhou X. (2013). MicroRNA-99 Family Members Suppress Homeobox A1 Expression in Epithelial Cells. PLoS ONE.

[76] Satoh M., Minami Y., Takahashi Y., Tabuchi T., Nakamura M. (2011). A Cellular MicroRNA, Let-7i, Is a Novel Biomarker for Clinical Outcome in Patients with Dilated Cardiomyopathy. J. Card. Fail..

[77] Miyamoto S.D., Karimpour-Fard A., Peterson V., Auerbach S.R., Stenmark K.R., Stauffer B.L., Sucharov C.C. (2015). Circulating MicroRNA as a Biomarker for Recovery in Pediatric Dilated Cardiomyopathy. J. Heart Lung Transplant..

[78] Tili E., Croce C.M., Michaille J.J. (2009). MiR-155: On the Crosstalk between Inflammation and Cancer. Int. Rev. Immunol..

[79] Seok H.Y., Chen J., Kataoka M., Huang Z.P., Ding J., Yan J., Hu X., Wang D.Z. (2014). Loss of MicroRNA-155 Protects the Heart from Pathological Cardiac Hypertrophy. Circ. Res..

[80] Liu J.J., Zhao C.M., Li Z.G., Wang Y.M., Miao W., Wu X.J., Wang W.J., Liu C., Wang D., Wang K. (2016). MiR-218 Involvement in Cardiomyocyte Hypertrophy Is Likely through Targeting REST. Int. J. Mol. Sci..

[81] Small E.M., Sutherland L.B., Rajagopalan K.N., Wang S., Olson E.N. (2010). Microrna-218 Regulates Vascular Patterning by Modulation of Slit-Robo Signaling. Circ. Res..

[82] Hassel D., Dahme T., Erdmann J., Meder B., Huge A., Stoll M., Just S., Hess A., Ehlermann P., Weichenhan D. (2009). Nexilin Mutations Destabilize Cardiac Z-Disks and Lead to Dilated Cardiomyopathy. Nat. Med..

[83] Hu Q.L., Xu Z.P., Lan Y.F., Li B. (2020). MiR-636 Represses Cell Survival by Targeting CDK6/Bcl-2 in Cervical Cancer. Kaohsiung J. Med. Sci..

[84] Misbah M., Kumar M., Lee K.H., Shen S.C. (2022). Identification of Novel MiRNAs, Targeting Genes, Signaling Pathway, and the Small Molecule for Overcoming Oxaliplatin Resistance of Metastatic Colorectal Cancer. BioMed Res. Int..

[85] Ma J., Zhou C., Chen X. (2021). MiR-636 Inhibits EMT, Cell Proliferation and Cell Cycle of Ovarian Cancer by Directly Targeting Transcription Factor Gli2 Involved in Hedgehog Pathway. Cancer Cell Int..

[86] Li L., Qiu X., Lv P., Wang F. (2014). MiR-639 Promotes the Proliferation and Invasion of Breast Cancer Cell In Vitro. Cancer Cell Int..

[87] Lei S., Shen F., Chen J., Feng J., Cai W., Shen L., Hu Z., Xu B. (2016). MiR-639 Promoted Cell Proliferation and Cell Cycle in Human Thyroid Cancer by Suppressing CDKN1A Expression. Biomed. Pharmacother..

[88] Li X., Lv J., Hou L., Guo X. (2022). Circ_0001955 Acts as a MiR-646 Sponge to Promote the Proliferation, Metastasis and Angiogenesis of Hepatocellular Carcinoma. Dig. Dis. Sci..

[89] Zhang P., Tang W.M., Zhang H., Li Y.Q., Peng Y., Wang J., Liu G.N., Huang X.T., Zhao J.J., Li G. (2017). MiR-646 Inhibited Cell Proliferation and EMT-Induced Metastasis by Targeting FOXK1 in Gastric Cancer. Br. J. Cancer.

[90] Jiao M., You H.Z., Yang X.Y., Yuan H., Li Y.L., Liu W.X., Jin M., Du J. (2018). Circulating MicroRNA Signature for the Diagnosis of Childhood Dilated Cardiomyopathy. Sci. Rep..

[91] Sharma S., Liu J., Wei J., Yuan H., Zhang T., Bishopric N.H. (2012). Repression of MiR-142 by P300 and MAPK Is Required for Survival Signalling via Gp130 during Adaptive Hypertrophy. EMBO Mol. Med..

[92] Liu B., Cheng M., Hu S., Wang S., Wang L., Tu X., Huang C., Jiang H., Wu G. (2018). Overexpression of MiR-142-3p Improves Mitochondrial Function in Cardiac Hypertrophy. Biomed. Pharmacother..

[93] Yu B., Zhao Y., Zhang H., Xie D., Nie W., Shi K. (2018). Inhibition of MicroRNA-143-3p Attenuates Myocardial Hypertrophy by Inhibiting Inflammatory Response. Cell Biol. Int..

[94] Ogawa K., Noda A., Ueda J., Ogata T., Matsuyama R., Nishizawa Y., Qiao S., Iwata S., Ito M., Fujihara Y. (2020). Forced Expression of MiR-143 and -145 in Cardiomyocytes Induces Cardiomyopathy with a Reductive Redox Shift. Cell. Mol. Biol. Lett..

[95] Deacon D.C., Nevis K.R., Cashman T.J., Zhou Y., Zhao L., Washko D., Guner-Ataman B., Burns C.G., Burns C.E. (2010). The MiR-143-Adducin3 Pathway Is Essential for Cardiac Chamber Morphogenesis. Development.

[96] Lozano-Velasco E., Galiano-Torres J., Jodar-Garcia A., Aranega A.E., Franco D. (2015). MiR-27 and MiR-125 Distinctly Regulate Muscle-Enriched Transcription Factors in Cardiac and Skeletal Myocytes. BioMed Res. Int..

[97] Hernandez-Torres F., Aranega A.E., Franco D. (2014). Identification of Regulatory Elements Directing MiR-23a-MiR-27a-MiR-24-2 Transcriptional Regulation in Response to Muscle Hypertrophic Stimuli. Biochim. Biophys. Acta Gene Regul. Mech..

[98] Zhou X., Sun F., Luo S., Zhao W., Yang T., Zhang G., Gao M., Lu R., Shu Y., Mu W. (2017). Let-7a Is an Antihypertrophic Regulator in the Heart via Targeting Calmodulin. Int. J. Biol. Sci..

[99] Jaguszewski M., Osipova J., Ghadri J.R., Napp L.C., Widera C., Franke J., Fijalkowski M., Nowak R., Fijalkowska M., Volkmann I. (2014). A Signature of Circulating MicroRNAs Differentiates Takotsubo Cardiomyopathy from Acute Myocardial Infarction. Eur. Heart J..

[100] Sonaglioni A., Nicolosi G.L., Rigamonti E., Lombardo M., La Sala L. (2022). Molecular Approaches and Echocardiographic Deformation Imaging in Detecting Myocardial Fibrosis. Int. J. Mol. Sci..

[101] Zimmerman M.S., Smith A.G.C., Sable C.A., Echko M.M., Wilner L.B., Olsen H.E., Atalay H.T., Awasthi A., Bhutta Z.A., Boucher J.L.A. (2020). Global, Regional, and National Burden of Congenital Heart Disease, 1990–2017: A Systematic Analysis for the Global Burden of Disease Study 2017. Lancet Child Adolesc. Health.

[102] Rohit M., Shrivastava S. (2018). Acyanotic and Cyanotic Congenital Heart Diseases. Indian J. Pediatr..

[103] Hu C., Huang S., Wu F., Ding H. (2020). MicroRNA-219-5p Participates in Cyanotic Congenital Heart Disease Progression by Regulating Cardiomyocyte Apoptosis. Exp. Ther. Med..

[104] Song Y., Higgins H., Guo J., Harrison K., Schultz E.N., Hales B.J., Moses E.K., Goldblatt J., Pachter N., Zhang G. (2018). Clinical Significance of Circulating MicroRNAs as Markers in Detecting and Predicting Congenital Heart Defects in Children. J. Transl. Med..

[105] Samani A., Hightower R.M., Reid A.L., English K.G., Lopez M.A., Scott Doyle J., Conklin M.J., Schneider D.A., Bamman M.M., Widrick J.J. (2022). MiR-486 Is Essential for Muscle Function and Suppresses a Dystrophic Transcriptome. Life Sci. Alliance.

[106] Lange S., Banerjee I., Carrion K., Serrano R., Habich L., Kameny R., Lengenfelder L., Dalton N., Meili R., Börgeson E. (2019). MiR-486 Is Modulated by Stretch and Increases Ventricular Growth. JCI Insight.

[107] Sun Y., Su Q., Li L., Wang X., Lu Y., Liang J. (2017). MiR-486 Regulates Cardiomyocyte Apoptosis by P53-Mediated BCL-2 Associated Mitochondrial Apoptotic Pathway. BMC Cardiovasc. Disord..

[108] Li D., Ji L., Liu L., Liu Y., Hou H., Yu K., Sun Q., Zhao Z. (2014). Characterization of Circulating MicroRNA Expression in Patients with a Ventricular Septal Defect. PLoS ONE.

[109] Seok H.Y., Tatsuguchi M., Callis T.E., He A., Pu W.T., Wang D.Z. (2011). MiR-155 Inhibits Expression of the MEF2A Protein to Repress Skeletal Muscle Differentiation. J. Biol. Chem..

[110] Chen W., Li S. (2017). Circulating MicroRNA as a Novel Biomarker for Pulmonary Arterial Hypertension Due to Congenital Heart Disease. Pediatr. Cardiol..

[111] Li X., Xiang D., Shu Y., Hu K., Zhang Y., Li Y. (2019). MicroRNA-204 as an Indicator of Severity of Pulmonary Hypertension in Children with Congenital Heart Disease Complicated with Pulmonary Hypertension. Med. Sci. Monit..

[112] Jin Y., Ai L., Chai X., Tang P., Zhang W., Yang L., Hu Y., Xu Y., Li S. (2021). Maternal Circulating Exosomal MiRNAs as Non-Invasive Biomarkers for the Prediction of Fetal Ventricular Septal Defect. Front. Genet..

[113] Zhu S., Cao L., Zhu J., Kong L., Jin J., Qian L., Zhu C., Hu X., Li M., Guo X. (2013). Identification of Maternal Serum MicroRNAs as Novel Non-Invasive Biomarkers for Prenatal Detection of Fetal Congenital Heart Defects. Clin. Chim. Acta.

[114] Yang Q., Wu F., Mi Y., Wang F., Cai K., Yang X., Zhang R., Liu L., Zhang Y., Wang Y. (2020). Aberrant Expression of MiR-29b-3p Influences Heart Development and Cardiomyocyte Proliferation by Targeting NOTCH2. Cell Prolif..

[115] Wang L., Song G., Liu M., Chen B., Chen Y., Shen Y., Zhu J., Zhou X. (2016). MicroRNA-375 Overexpression Influences P19 Cell Proliferation, Apoptosis and Differentiation through the Notch Signaling Pathway. Int. J. Mol. Med..

[116] Huang Z.P., Wang D.Z. (2014). MiR-22 in Cardiac Remodeling and Disease. Trends Cardiovasc. Med..

[117] Gu H., Chen L., Xue J., Huang T., Wei X., Liu D., Ma W., Cao S., Yuan Z. (2019). Expression Profile of Maternal Circulating MicroRNAs as Non-Invasive Biomarkers for Prenatal Diagnosis of Congenital Heart Defects. Biomed. Pharmacother..

[118] You G., Zu B., Wang B., Fu Q., Li F. (2020). Identification of MiRNA–MRNA–TFs Regulatory Network and Crucial Pathways Involved in Tetralogy of Fallot. Front. Genet..

[119] Zhang J., Qin L., Han L., Zhao Y., Jing H., Song W., Shi H. (2017). Role of MicroRNA-93 I in Pathogenesis of Left Ventricular Remodeling via Targeting Cyclin-D1. Med. Sci. Monit..

[120] Wo Y., Guo J., Li P., Yang H., Wo J. (2018). Long Non-Coding RNA CHRF Facilitates Cardiac Hypertrophy through Regulating Akt3 via MiR-93. Cardiovasc. Pathol..

[121] Ektesabi A.M., Mori K., Tsoporis J., Walsh C., Mai S., Hu P., DosSantos C. (2019). Regulation of Mir-187b in Endotoxemic Primary Cardiomyocytes and Septic Murine Hearts Treated with Mesenchymal Stromal/Stem Cells. Can. J. Cardiol..

[122] Zhang J., Chang J.J., Xu F., Ma X.J., Wu Y., Li W.C., Wang H.J., Huang G.Y., Ma D. (2013). MicroRNA Deregulation in Right Ventricular Outflow Tract Myocardium in Nonsyndromic Tetralogy of Fallot. Can. J. Cardiol..

[123] Grunert M., Appelt S., Dunkel I., Berger F., Sperling S.R. (2019). Altered MicroRNA and Target Gene Expression Related to Tetralogy of Fallot. Sci. Rep..

[124] Wang B., Shi G., Zhu Z., Chen H., Fu Q. (2018). Sexual Difference of Small RNA Expression in Tetralogy of Fallot. Sci. Rep..

[125] Bittel D.C., Kibiryeva N., Marshall J.A., O’Brien J.E. (2014). MicroRNA-421 Dysregulation Is Associated with Tetralogy of Fallot. Cells.

[126] Wu K.H., Xiao Q.R., Yang Y., Xu J.L., Zhang F., Liu C.M., Zhang Z.M., Lu Y.Q., Huang N.P. (2018). MicroRNA-34a Modulates the Notch Signaling Pathway in Mice with Congenital Heart Disease and Its Role in Heart Development. J. Mol. Cell. Cardiol..

[127] Sánchez-Gómez M.C., García-Mejía K.A., Pérez-Díaz Conti M., Díaz-Rosas G., Palma-Lara I., Sánchez-Urbina R., Klünder-Klünder M., Botello-Flores J.A., Balderrábano-Saucedo N.A., Contreras-Ramos A. (2017). MicroRNAs Association in the Cardiac Hypertrophy Secondary to Complex Congenital Heart Disease in Children. Pediatr. Cardiol..

[128] Zloto K., Tirosh-Wagner T., Bolkier Y., Bar-Yosef O., Vardi A., Mishali D., Paret G., Nevo-Caspi Y. (2020). Preoperative MiRNA-208a as a Predictor of Postoperative Complications in Children with Congenital Heart Disease Undergoing Heart Surgery. J Cardiovasc. Transl. Res..

[129] Huang J., Li X., Li H., Su Z., Wang J., Zhang H. (2015). Down-Regulation of MicroRNA-184 Contributes to the Development of Cyanotic Congenital Heart Diseases. Int. J. Clin. Exp. Pathol..

[130] Zhang Y., Peng B., Han Y. (2018). MiR-182 Alleviates the Development of Cyanotic Congenital Heart Disease by Suppressing HES1. Eur. J. Pharmacol..

[131] Zhou Y., Jia W.K., Jian Z., Zhao L., Liu C.C., Wang Y., Xiao Y. (2017). Bin Downregulation of MicroRNA-199a-5p Protects Cardiomyocytes in Cyanotic Congenital Heart Disease by Attenuating Endoplasmic Reticulum Stress. Mol. Med. Rep..

[132] Sucharov C.C., Sucharov J., Karimpour-Fard A., Nunley K., Stauffer B.L., Miyamoto S.D. (2015). Micro-RNA Expression in Hypoplastic Left Heart Syndrome. J. Card. Fail..

[133] Smolka C., Schlösser D., Koentges C., Tarkhnishvili A., Gorka O., Pfeifer D., Bemtgen X., Asmussen A., Groß O., Diehl P. (2021). Cardiomyocyte-Specific MiR-100 Overexpression Preserves Heart Function under Pressure Overload in Mice and Diminishes Fatty Acid Uptake as Well as ROS Production by Direct Suppression of Nox4 and CD36. FASEB J..

[134] Wong L.L., Wee A.S.Y., Lim J.Y., Ng J.Y.X., Chong J.P.C., Liew O.W., Lilyanna S., Martinez E.C., Ackers-Johnson M.A., Vardy L.A. (2015). Natriuretic Peptide Receptor 3 (NPR3) Is Regulated by MicroRNA-100. J. Mol. Cell. Cardiol..

[135] Chen A., Li G., Chen L., Guo J., Liu Y. (2015). Downregulation of MicroRNA-100 Protects H_2_O_2_-Induced Apoptosis in Neonatal Cardiomyocytes. Int. J. Clin. Exp. Pathol..

[136] Wang L., Tian D., Hu J., Xing H., Sun M., Wang J., Jian Q., Yang H. (2016). MiRNA-145 Regulates the Development of Congenital Heart Disease Through Targeting FXN. Pediatr. Cardiol..

[137] Borghini A., Vecoli C., Mercuri A., Turchi S., Andreassi M.G. (2021). Individual and Joint Effects of Genetic Polymorphisms in MicroRNA-Machinery Genes on Congenital Heart Disease Susceptibility. Cardiol. Young.

[138] Toro R., Pérez-Serra A., Mangas A., Campuzano O., Sarquella-Brugada G., Quezada-Feijoo M., Ramos M., Alcalá M., Carrera E., García-Padilla C. (2022). MiR-16-5p Suppression Protects Human Cardiomyocytes against Endoplasmic Reticulum and Oxidative Stress-Induced Injury. Int. J. Mol. Sci..

[139] Huang C.Y., Pai P.Y., Kuo C.H., Ho T.J., Lin J.Y., Lin D.Y., Tsai F.J., Padma V.V., Kuo W.W., Huang C.Y. (2017). P53-Mediated MiR-18 Repression Activates HSF2 for IGF-IIR-Dependent Myocyte Hypertrophy in Hypertension-Induced Heart Failure. Cell Death Dis..

[140] Huang G.-J., Xie X.-L., Zou Y. (2022). MiR-23b Targets GATA6 to down-Regulate IGF-1 and Promote the Development of Congenital Heart Disease. Acta Cardiol..

[141] Icli B., Dorbala P., Feinberg M.W. (2014). An Emerging Role for the MiR-26 Family in Cardiovascular Disease. Trends Cardiovasc. Med..

[142] Yuan R., Zhang X., Fang Y., Nie Y., Cai S., Chen Y., Mo D. (2018). Mir-127-3p Inhibits the Proliferation of Myocytes by Targeting KMT5a. Biochem. Biophys. Res. Commun..

[143] Li J., Wang G., Jiang J., Zhang L., Zhou P., Ren H. (2020). MicroRNA-127-3p Regulates Myoblast Proliferation by Targeting Sept7. Biotechnol. Lett..

[144] Wang L., Qin D., Shi H., Zhang Y., Li H., Han Q. (2019). MiR-195-5p Promotes Cardiomyocyte Hypertrophy by Targeting MFN2 and FBXW7. BioMed Res. Int..

[145] Cheng X., Du J., Shen L., Tan Z., Jiang D., Jiang A., Li Q., Tang G., Jiang Y., Wang J. (2018). MiR-204-5p Regulates C2C12 Myoblast Differentiation by Targeting MEF2C and ERRγ. Biomed. Pharmacother..

[146] Xuan Y., Liu S., Li Y., Dong J., Luo J., Liu T., Jin Y., Sun Z. (2017). Short-Term Vagus Nerve Stimulation Reduces Myocardial Apoptosis by Downregulating MicroRNA-205 in Rats with Chronic Heart Failure. Mol. Med. Rep..

[147] Salant G.M., Tat K.L., Goodrich J.A., Kugel J.F. (2020). MiR-206 Knockout Shows It Is Critical for Myogenesis and Directly Regulates Newly Identified Target MRNAs. RNA Biol..

[148] Anderson C., Catoe H., Werner R. (2006). MIR-206 Regulates Connexin43 Expression during Skeletal Muscle Development. Nucleic Acids Res..

[149] Zhao X., Wang Y., Sun X. (2020). The Functions of MicroRNA-208 in the Heart. Diabetes Res. Clin. Pract..

[150] Xu F., Yang J., Shang J., Lan F., Li M., Shi L., Shen L., Wang Y., Ge J. (2019). MicroRNA-302d Promotes the Proliferation of Human Pluripotent Stem Cell-Derived Cardiomyocytes by Inhibiting LATS2 in the Hippo Pathway. Clin. Sci..

[151] Fang Y.C., Yeh C.H. (2017). Inhibition of MIR-302 Suppresses Hypoxia-Reoxygenation-Induced H9c2 Cardiomyocyte Death by Regulating Mcl-1 Expression. Oxid. Med. Cell. Longev..

[152] Li Y., Li X., Wang L., Han N., Yin G. (2021). MiR-375-3p Contributes to Hypoxia-Induced Apoptosis by Targeting Forkhead Box P1 (FOXP1) and Bcl2 like Protein 2 (Bcl2l2) in Rat Cardiomyocyte H9c2 Cells. Biotechnol. Lett..

[153] Feng H., Wu J., Chen P., Wang J., Deng Y., Zhu G., Xian J., Huang L., Ouyang W. (2019). MicroRNA-375-3p Inhibitor Suppresses Angiotensin II-Induced Cardiomyocyte Hypertrophy by Promoting Lactate Dehydrogenase B Expression. J. Cell. Physiol..

[154] Knezevic I., Patel A., Sundaresan N.R., Gupta M.P., Solaro R.J., Nagalingam R.S., Gupta M. (2012). A Novel Cardiomyocyte-Enriched MicroRNA, MiR-378, Targets Insulin-like Growth Factor 1 Receptor: Implications in Postnatal Cardiac Remodeling and Cell Survival. J. Biol. Chem..

[155] Yuan J., Liu H., Gao W., Zhang L., Ye Y., Yuan L., Ding Z., Wu J., Kang L., Zhang X. (2018). MicroRNA-378 Suppresses Myocardial Fibrosis through a Paracrine Mechanism at the Early Stage of Cardiac Hypertrophy Following Mechanical Stress. Theranostics.

[156] Fang J., Song X.W., Tian J., Chen H.Y., Li D.F., Wang J.F., Ren A.J., Yuan W.J., Lin L. (2012). Overexpression of MicroRNA-378 Attenuates Ischemia-Induced Apoptosis by Inhibiting Caspase-3 Expression in Cardiac Myocytes. Apoptosis.

[157] Matkovich S.J., Hu Y., Dorn G.W. (2013). Regulation of Cardiac MicroRNAs by Cardiac MicroRNAs. Circ. Res..

[158] Ling T.Y., Wang X.L., Chai Q., Lau T.W., Koestler C.M., Park S.J., Daly R.C., Greason K.L., Jen J., Wu L.Q. (2013). Regulation of the SK3 Channel by MicroRNA-499—Potential Role in Atrial Fibrillation. Heart Rhythm.

[159] Wang J., Jia Z., Zhang C., Sun M., Wang W., Chen P., Ma K., Zhang Y., Li X., Zhou C. (2014). MiR-499 Protects Cardiomyocytes from H2O2-Induced Apoptosis via Its Effects on Pdcd4 and Pacs2. RNA Biol..

[160] Shi Y., Han Y., Niu L., Li J., Chen Y. (2019). MiR-499 Inhibited Hypoxia/Reoxygenation Induced Cardiomyocytes Injury by Targeting SOX6. Biotechnol. Lett..

[161] Matkovich S.J., Hu Y., Eschenbacher W.H., Dorn L.E., Dorn G.W. (2012). Direct and Indirect Involvement of MicroRNA-499 in Clinical and Experimental Cardiomyopathy. Circ. Res..

[162] Yang H., Su J., Meng W., Chen X., Xu Y., Sun B. (2021). Mir-518a-5p Targets Gzmb to Extenuate Vascular Endothelial Cell Injury Induced by Hypoxia-Reoxygenation and Thereby Improves Myocardial Ischemia. Int. Heart J..

[163] Kang T., Xing W., Xi Y., Chen K., Zhan M., Tang X., Wang Y., Zhang R., Lei M. (2020). MiR-543 Regulates Myoblast Proliferation and Differentiation of C2C12 Cells by Targeting KLF6. J. Cell. Biochem..

[164] Yang M., Liu X., Jiang M., Li J., Tang Y., Zhou L. (2021). MiR-543 in Human Mesenchymal Stem Cell–Derived Exosomes Promotes Cardiac Microvascular Endothelial Cell Angiogenesis after Myocardial Infarction through COL4A1. IUBMB Life.

[165] Nymark P., Wijshoff P., Cavill R., Van Herwijnen M., Coonen M.L.J., Claessen S., Catalán J., Norppa H., Kleinjans J.C.S., Briedé J.J. (2015). Extensive Temporal Transcriptome and MicroRNA Analyses Identify Molecular Mechanisms Underlying Mitochondrial Dysfunction Induced by Multi-Walled Carbon Nanotubes in Human Lung Cells. Nanotoxicology.

