# Supplementary material for: Role of miRNA in Cardiovascular Diseases in Children—Systematic Review
Source: Int J Mol Sci. 2024 Jan 12;25(2):956. doi: 10.3390/ijms25020956 (PMC10816020; doi:10.3390/ijms25020956)
Supplement: Supplementary file 1 [file ijms-25-00956-s001.zip › ijms-2753675-supplementary.pdf]

International Journal of
Molecular Sciences

MDPI

Table S1: miRNA and their role in cardiovascular diseases

miRNA	Role	Targets	Chromosomes*	references
**miR-1**	• regulation in arrhythmias debatable<br>• has good sensitivity and specificity for evaluation of SVT<br>• regulates cardiac conduction, cardiac automaticity, cardiac repolarization, impacts calcium handling<br>• was proved to induce atrioventricular block due to inhibiting potassium channel (Kir2.1) expression in mice<br>• downregulated in VCM, negatively correlated with left ventricular FS and LVEF in children with VCM<br>• strongly associated with cell damage in children with cardiac hypertrophy<br>• downregulated in VSD and ToF<br>• might be responsible for variable expression of small RNA (sRNA) between sexes in ToF	*Ap1s1*<br>*Bax*<br>*Braf*<br>*CaM*<br>*Casp9*<br>*Ccz1*<br>*GJA*<br>*KCNJ2*<br>*Kcnj2*<br>*Kcnd2*<br>*Mapk8ip3*<br>*Mef2*<br>*SOX9*<br>*Stx6*<br>*Ube3a*	miR-1-1 chr20<br>miR-1-2 chr18	[1–12]
**miR-let-7**	• Let-7 family regulates cardiomyocyte regeneration, cell survival, metabolic control and glucose utilization<br>• miR-let-7a and miR-let-7b is specifically related to ASD but not other subtypes of septal defects in children<br>• higer levels of miR-let-7a, miR-let-7b were observed in parents of children with CHD<br>• miR-let-7a was increased in patients with familial DCM caused by Lamin A/C (LMNA) gene mutations and seems to possess anti-hypertrophic property by targeting calmodulin genes	*ABCB9*<br>*ACTA1*<br>*ANKRA2*<br>*ANKRD49*<br>*BRF2*<br>*CaM*<br>*CLP1*<br>*Ezh2*<br>*GATA3*<br>*HAND1*<br>*NKD1*<br>*NME6*<br>*NOTCH1*<br>*XKR8*<br>*ZFPM2*	hsa-let-7a-1 chr9<br>hsa-let-7a-2 chr11<br>hsa-let-7a-3 chr22<br>hsa-let-7b chr22<br>hsa-let-7c chr21<br>hsa-let-7d chr9<br>hsa-let-7e chr19<br>hsa-let-7f-1 chr9<br>hsa-let-7f-2 chrX<br>hsa-let-7g chr3<br>hsa-let-7i chr12	[13–18]

	• miR-let-7e-5p is downregulated in VSD and targets genes related to cardiac development<br>• let-7f-5p, let-7g-5p are upregulated in DCM, negative correlation was obserwed between let-7g-5p expression and ejection fraction which might suggest its role as a potential biomarker for the assessment of heart failure in DCM<br>• decrease of miR-7i is related with poor clinical outcomes in patients with DCM	*TRIM71*<br>*ZNF583*<br>*NEK3*<br>*AURKB*<br>*SLC12A9*<br>*SMUG1*<br>*ADRB2*<br>*SLC35D2*<br>*UFM1*<br>*EZH2*<br>*TIMM17B*<br>*GPR137*<br>*ZNF354A*<br>*ATP2A2*<br>*NME4*<br>*BZW2*<br>*LIPH*<br>*DTX2*<br>*BACE2*<br>*DHX57*<br>*TARBP2*<br>*GALE*<br>*MDFI*<br>*RPUSD3*<br>*CCL7*<br>*OLFM4*<br>*TRAPPC1*<br>*KIAA1539*<br>*RNF20*<br>*KLHDC8B*<br>*FAM103A1*<br>*PRTG*<br>*BZW1*<br>*GNPTAB*<br>*COIL*<br>*AP1S1*<br>*HIF3A*<br>*TTLL6*<br>*TNFSF9*<br>*PLEKHG6*<br>*GNG5*<br>*C8orf58*		

		GATM<br>*B3GNT1*<br>*CTHRC1*<br>*TGDS*<br>*GATA4*<br>*RTKN*<br>*USP21*<br>*LRIG2*<br>*AVEN*<br>*PRSS22*<br>*ZC3H3*<br>*KLHL6*<br>*ABCC10*<br>*YTHDF3*<br>*STXBP5*<br>*PLEKHO1*<br>*DDX19B*<br>*NID2*<br>*IL13*<br>*FASLG*<br>*PSORS1C2*<br>*NTRK3*<br>*MED28*<br>*LD3*<br>*CDC34*<br>*MGAT4A*<br>*SPATA2*<br>*LOR*<br>*ISLR*<br>*LRIG3*<br>*ABCC5*<br>*POLL*<br>*NDST2*<br>*CDKN1A*<br>*CRCT1*<br>*CTNS*<br>*THRSP*<br>*CHRD*<br>*ZNF341*<br>*KCTD16*<br>*QARS*<br>*PQLC2*		

		ESPL1<br>*SLC20A1*		
**miR-16**	• downregulated in children with rheumatic carditis<br>• lower levels may play a preventive role against inflammation	*ATF6*	hsa-mir-16-1 chr13<br>hsa-mir-16-2 chr3	[19,20]
**miR-17**	• regulation in hypertrophy debatable<br>• exhibits sex-specific difference in expression<br>• inhibits autophagy and promotes pathological cardiac hypertrophy	*Mfn2*<br>*ICAM-1*	chr13	[21–23]
**miR-18**	• upregulated in cardiac hypertrophy caused by CHD	*HSF2*	hsa-mir-18a chr13<br>hsa-mir-18b chrX	[11,24]
**miR-19**	• miR-19a may be a novel biomarker for diagnosis of PAH<br>• miR-19b upregulated in ASD and VSD and might also be used as a prenatal marker of CHD in fetus<br>• miR-19b regulation in ToF debatable	*FGFR2*	hsa-mir-19a chr13<br>hsa-mir-19b-1 chr13<br>hsa-mir-19b-2 chrX	[25,26]
**miR-21**	• cardiac-associated and inflammatory related miRNA<br>• downregulated in the resolution/chronic phase of myocarditis<br>• upregulated in cardiac hypertrophy caused by CHD, may be considered as specific biomarker for the diagnosis of cardiac hypertrophy in infants with CHD	*Bcl-2*<br>*CDK6*<br>*KBTBD7*<br>*Rcan1*<br>*SORBS2*	hsa-mir-21 chr17	[11,27–32]
**miR-22**	• targets genes involved in hypertrophy development and may be specifically up-regulated in ToF<br>• might be used as a prenatal marker of CHD in fetus	*HDAC4*<br>*PGC1-α*<br>*PPARα*<br>*PurB*<br>*Sirt1*	chr17	[26,33]
**miR-23**	• participates in cardiac development,<br>• miR-23b upregulated in ToF, can be considered as specific biomarker for the diagnosis of cardiac hypertrophy in infants with CHD.	*GATA6*	hsa-mir-23a chr19<br>hsa-mir-23b chr9<br>hsa-mir-23c chrX	[34,35]
**miR-26**	• upregulated in DCM<br>• mir-26a is significantly increased in Takotsubo cardiomyopathy patients	*GSK3β*<br>*KCNJ2*<br>*PLCβ1*	hsa-mir-26a-1 chr3<br>hsa-mir-26a-2 chr12<br>hsa-mir-26b chr2	[14,36,37]

	compared with its levels in healthy adults			
**miR-27**	• miR-27a and miR-27b are upregulated in DCM, miR-27b may become useful tool in childhood DCM detection and diagnosis<br>• miR-23a–miR-27a–miR-24-2 cluster might response to angiotensin and norepinephrine-driven prohypertrophic signaling pathways<br>• may become promising molecular target for reversing the remodeling seen in PAH<br>• may exhibit proagniogenic properties	*Mstn*<br>*Myocd*<br>*Mdfi*	hsa-mir-27a chr19<br>hsa-mir-27b chr9	[14,23,25,38]
**miR-29**	• miR-29 family suppresses excess collagen expression, it may also promote cardiac hypertrophy and CHD development<br>• miR-29a is upregulated in ToF<br>• miR-29b inhibits cardiomyocyte proliferation<br>• miR-29c correlates especially with VSD, regulation in ToF debatable	*Akt3*<br>*NOTCH2*	hsa-mir-29a chr7<br>hsa-mir-29b-1 chr7<br>hsa-mir-29b-2 chr1<br>hsa-mir-29c chr1	[12,26,39]
**miR-34**	• miR-34a regulates proliferation and differentiation of cells, in endothelial cells it decreases Sirtuin 1 gene, that protects cells against oxidative and genotoxic stress<br>• miR-34a participates in the pathogenesis of CHD in mice	*NOTCH-1*<br>*SIRT1*	hsa-mir-34a chr1<br>hsa-mir-34b chr11<br>hsa-mir-34c chr11	[23,40]
**miR-92**	• downregulated in children with rheumatic carditis<br>• decrease in miR-92a may play a protective role against ischemia and tissue necrosis as shown in animal studies.		hsa-mir-92a-1 chr13<br>hsa-mir-92a-2 chrX<br>hsa-mir-92b chr1	[19,41]
**miR-93**	• upregulated in ToF, suppresses cardiac hypertrophy responses<br>• targets cyclin D1 gene *CCND1*, disruption in miR-93/CCND1 signaling pathway was responsible for the development of ventricular remodeling	*CCDN1*	chr7	[42,43]

**miR-99**	• downregulated in the DCM<br>• controls cardiomyogenesis and targets genes responsible for regulating epithelial cell proliferation and migration<br>• overexpression of miR-99 may diverge the physiological hypertrophy to pathological hypertrophy by regulating Akt-1 pathway<br>• significant change was observed in miR-99 expression level in correlation with the stage of surgery in HLHS patients, might suggest that volume unloading of the ventricle has important consequences for gene expression	*CTDSPL*<br>*HOXA1*<br>*SMARCA5*<br>*mTOR*<br>*NMT1*<br>*TMEM30A*	hsa-mir-99a chr21<br>hsa-mir-99b chr19	[44–48]
**miR-100**	• significant change was observed in miR-100 expression level with the stage of surgery of HLHS which might suggest that volume unloading of the ventricle has important consequences for gene expression<br>• upregulated and protected mice heart subjected from pressure overload<br>• regulates cardiomyocyte hypoxia-induced apoptosis by suppressing the expression of insulin-like growth factor 1 receptor (*IGF1R*)	*IGF1R*<br>*Nox4*<br>*NPR3*	chr11	[48–50]
**miR-107**	• upregulated in DCM, exhibits sex-specific difference in expression		chr10	[21]
**miR-126**	• involved in vascular dysfunction and modifies vascular inflammation<br>• upregulated in DCM, might become a useful tool in childhood DCM detection and diagnosis, negative correlation between miR-126 expression and ejection fraction was observed	*EGFL7*<br>*PIK3R2*<br>*SPRED1*<br>*VCAM-1*	chr9	[14,23]
**miR-127**	• upregulated in ToF, heart and muscle-related<br>• regulates profileration of myoblasts and myocytes	*KMT5a*<br>*Sept7*	chr14	[12,51,52]
**miR-130**	• miR-130a exhibits proangiogenic properties	*GAX*<br>*HOXA5*	hsa-mir-130a chr11<br>hsa-mir-130b chr22	[23,25]

**miR-133**	• plays important role in promoting differentiation of fibroblasts into cardiomyocyte-like cells<br>• targets potassium channels and controls cardiac repolarization<br>• miR-133a moderates the expression of beta-myosin heavy chains (β-MHC) in children with CH.<br>• miR-133b is downregulated in VCM, negatively correlates with myocardial injuries, targets *Rab27b* which promotes injuries of cardiomyocytes induced by CVB3 infection and facilitates the synthesis and release of cytokines TNF-α and IL-6, reduces cardiomyocyte apoptosis by affecting apoptosis-related genes<br>• miR-133b is downregulated in ToF<br>• might be responsible for variable expression of small RNA (sRNA) between sexes in ToF	*FBN2*<br>*KCNE1*<br>*KCNQ1*<br>*HCN2*<br>*HCN4*<br>*Rab27b*<br>*SLC38A3*<br>*TNNI1*	hsa-mir-133a-1 chr18<br>hsa-mir-133a-2 chr20<br>hsa-mir-133b chr6	[1,7,10,12,53–55]
**miR-142**	• upregulated in DCM, might become a useful tool in childhood DCM detection and diagnosis<br>• protects mitochondrial function and inhibits the expression of *SH2B1* gene which directly leads to alleviation of cardiac hypertrophy<br>• may be used to distinguish fetuses with VSD, ToF, SV and PTA from the healthy ones	*SH2B1*	chr17	[14,56–58]
**miR-143**	• essential for cardiac chamber morphogenesis<br>• upregulated in hypertrophy models, attenuated inflammatory response induced by myocardial hypertrophy<br>• upregulated in DCM, might become a useful tool in childhood DCM detection and diagnosis	*ERK5*<br>*Add3*<br>*HK2*	chr5	[14,59–61]
**miR-145**	• influences the apoptosis and mitochondrial function<br>• regulates the development of CHD	*FXN*	chr5	[62]

**miR-146**	• miR-146a5 regulates development and morphogenesis of the heart muscle and may potentially be used as a biomarker for prenatal detection of VSD, downregulated in VSD<br>• miR-146b is an inflammation-related miRNA, upregulated in VCM, serum levels of miR-146b were positively correlated with left ventricular FS and LVEF in VCM,<br>• inhibition of miR-146b reduces inflammatory lesions and suppresses Th-17 differentiation therefore leading to relief in the severity of myocarditis<br>• represses endothelial activation by inhibiting pro-inflammatory pathways, protects cardiomyocytes from injury during ischemia and can be downregulated by hypoxia	*CCL5*<br>*ERBB4*<br>*IRAK1*<br>*IRAK2*<br>*NUMB*<br>*PMAIP1*<br>*TRAF6*	hsa-mir-146a chr5<br>hsa-mir-146b chr10	[8,23,63–67]
**miR-147**	• downregulated in DCM<br>• lower levels of miR-147 led to increase of inflammation in myocardium<br>• targets and inhibits hyperpolarization activated cyclic nucleotide-gated potassium channel 4 (*HCN4*) gene expression which upregulation is supposed to be responsible for causing heart failure and ischemic cardiomyopathy	*HCN4*	hsa-mir-147a chr9<br>hsa-mir-147b chr15	[47]
**miR-155**	• downregulated in DCM<br>• downregulated in VSD, regulates *MEF2A* which deficiency in mice caused dilation of the right ventricle, myofibrillar fragmentation, mitochondrial disorganization and activation of a fetal cardiac gene program and death in consequence<br>• upregulated in ToF, participates in cardiac development<br>• plays role in initiating endothelial dysfunction, structural remodeling, and vascular inflammation	*ACTA1*<br>*ACTR10*<br>*AGTRAP*<br>*AICDA*<br>*ARRB2*<br>*AT1R*<br>*BAIAP2L1*<br>*BCORL1*<br>*BOC*<br>*BRD1*<br>*CARHSP1*<br>*CDC73*<br>*CEBPB*	chr21	[15,18,34,47]

		CHD7<br>*CLCN5*<br>*COL21A1*<br>*CSF1R*<br>*CSNK1G2*<br>*C8orf4*<br>*DCLRE1A*<br>*DET1*<br>*DHX40*<br>*DNAJB7*<br>*DYNC1I1*<br>*FAM105A*<br>*FBXO11*<br>*FOS*<br>*GNAS*<br>*HBP1*<br>*HIVEP2*<br>*H3F3A*<br>*IKBKE*<br>*IL13*<br>*JARID2*<br>*KIAA1267*<br>*KIAA1715*<br>*LAMP2*<br>*LRP1B*<br>*LSM14A*<br>*MAP3K10*<br>*MBNL3*<br>*MEF2A*<br>*MGP*<br>*MYLK*<br>*MYO10*<br>*PCDH9*<br>*PDE7A*<br>*PHC2*<br>*PSIP1*<br>*PTPN2*<br>*RCN2*<br>*RNF123*<br>*RNF149*<br>*SALL1*<br>*SAP30L*		

		SDCBP<br>*SGK3*<br>*SHOX*<br>*SKIV2L2*<br>*SLC12A6*<br>*SMARCA4*<br>*SOCS1*<br>*SPI1*<br>*SPIN3*<br>*STXBP5L*<br>*TAPT1*<br>*TBX-1*<br>*TERF1*<br>*TPRKB*<br>*TRIM32*<br>*USP8*<br>*USP43*<br>*WDR45*<br>*WEE1*<br>*VPS18*<br>*ZBTB38*		
**miR-182**	• alleviates CHD development due to suppressing hairy and enhancer of split-1	*HES1*	chr7	[68]
**miR-184**	• downregulated in patients with cyanotic congenital heart disease<br>• inhibition of miR-184 caused decrease in cell viability and induction of apoptosis under hypoxia, levels of apoptotic proteins caspase-3 and caspase-9 significantly increased due to miR-184 inhibition		chr15	[69]
**miR-187**	• targets genes responsible for regulating cardiomyocyte apoptosis and cardiac inflammation<br>• upregulated in ToF	*Itpkc,*<br>*Lrrc59*<br>*Tbl1xr1*	chr18	[34,70]
**miR-194**	• downregulated in DCM, targets heparin-binding EGF-like growth factor (*HBEGF*) gene which impairs phosphorylation of ERBB2/B4 tyrosine kinase receptors and leads to severe DCM	*HBEGF*	hsa-mir-194-1 chr1<br>hsa-mir-194-2 chr11	[47,71,72]
**miR-195**	• promotes cardiac hypertrophy	*FBXW7*	chr17	[11,73]

	• upregulated in cardiac hypertrophy caused by CHD	*MFN2*		
**miR-199**	• miR-199a-5p attenuates endoplasmic reticulum stress in cyanotic CHD	*ATF6*<br>*GRP78*	hsa-mir-199a-1 chr19<br>hsa-mir-199a-2 chr1<br>hsa-mir-199b chr9	[74]
**miR-204**	• regulates myoblast differentiation<br>• involved in pulmonary hypertension development in children with CHD	*ERRγ*<br>*MEF2C*	chr9	[75,76]
**miR-205**	• downregulated in DCM, targets myocardial zonula adherens protein gene which knockdown is related to DCM,<br>• relates with angiogenesis promotion and cardiomyocyte apoptosis inhibition<br>• involved in the induction of inflammation and atherosclerosis in vascular endothelial cells	*MYZAP*	chr1	[47,77–79]
**miR-206**	• important for myogenesis<br>• upregulated in ToF, heart and muscle-related	*Adam19*<br>*Bgn*<br>*Cbx5*<br>*Cx43*<br>*Smarce1*<br>*Spg20*	chr6	[12,80,81]
**miR-208**	• expressed by cardiomyocytes and released upon myocardial damage<br>• miR-208a was upregulated during the acute phase of enteroviral, adenoviral or parvoviral B19 myocarditis<br>• miR-208a was significantly decreased during both the subacute phase and the resolution/chronic phase which correlated with the significantly downregulated levels of cardiac- and inflammatory associated miR-21 during the chronic/resolution phase of enteroviral, adenoviral or parvoviral B19 myocarditis<br>• level of miR-208b did not change significantly during the subacute and resolution/ chronic phases of enteroviral, adenoviral or parvoviral B19 myocarditis	*GATA4*<br>*HP1B*<br>*Med13*<br>*Mstn*<br>*PurB*<br>*Sox6*<br>*SP3*<br>*Thrap1*	chr14	[21,27,28,32,82–86]

	• miR-208a may be used as a diagnostic marker of cardiac damage and miR-208b as a prognostic marker for left ventricular function recovery in children with myocarditis<br>• miR-208a was downregulated in DCM,<br>• miR-208a targets *Thrap1* and myostatin which are important negative regulators of muscle growth and hypertrophy, might be used as a biomarker of postoperative complications in pediatric patients with CHD that underwent surgery<br>• miR-208b is upregulated in cardiac hypertrophy caused by CHD			
**miR-217**	• expressed in human aortic endothelial cells and human coronary artery endothelial cells, may exhibit proangiogenic properties<br>• upregulated in viral myocarditis, its inhibition attenuates viral myocarditis by inhibiting the apoptosis of cardiomyocytes and preventing the inflammatory response and oxidative stress by targeting *SIRT1*	*SIRT1*	chr2	[23,87]
**miR-218**	• downregulated in DCM, regulates RE1-silencing transcription factor (*REST*) and its suppression influence cardiomyocytes hypertrophy development if suppressed	*NEXN*<br>*REST*	hsa-mir-218-1 chr4<br>hsa-mir-218-2 chr5	[47,88]
**miR-219**	• upregulated in cyanotic congenital heart disease but not in acyanotic CHD,<br>• the level of miR-219 increases gradually in hypoxic conditions in a time-dependent manner, its downregulation may inhibit hypoxia-induced cardiomyocyte apoptosis	*LRH-1*	hsa-mir-219a-1 chr6<br>hsa-mir-219a-2 chr9<br>hsa-mir-219b chr9	[89]
**miR-222**	• antiangiogenic factor<br>• downregulated in VSD, targets genes related to cardiac development<br>• upregulated in ventricular outflow tract tissues obtained from infants with non-	*ADAMTS6*<br>*ANKRD10*<br>*AP3B2*<br>*CCDC64*<br>*CDKN1B*	chrX	[18,23,34,90]

		syndromic ToF, increases cell proliferation and inhibits cardiomyogenic differentiation	*CDKN1C*<br>*CYR61*<br>*c-Kit*<br>*DMRT3*<br>*eNOS*<br>*FNDC3A*<br>*FOS*<br>*FRAT2*<br>*GATA3*<br>*GRM1*<br>*GNAI2*<br>*HAND1*<br>*HEXIM1*<br>*HOXC10*<br>*IRX5*<br>*KHDRBS2*<br>*KIAA1370*<br>*KRT81*<br>*KSR1*<br>*LRFN2*<br>*MAPK10*<br>*MBD2*<br>*MESDC1*<br>*MIA3*<br>*MIDN*<br>*NOTCH1*<br>*NSMCE4A*<br>*NTF3*<br>*PAIP2*<br>*PANK3*<br>*PBX3*<br>*PCDHA4*<br>*PCMTD1*<br>*PLCL2*<br>*RBM24*<br>*RECK*<br>*RSBN1L*<br>*SEMA3B*<br>*SMARCA5*<br>*SNCB*<br>*TBX1*<br>*TCF7L2*		

		TP53BP2<br>*TOX*<br>*VGLL4*<br>*YTHDC1*<br>*ZFPM2*<br>*ZFYVE16*<br>*ZNF181*		
**miR-223**	• acts as a classical immunoregulatory gene<br>• downregulated in children with rheumatic carditis<br>• regulates immune cell functions by reducing inflammation, may protect from heart chamber dilatation<br>• might be used in the diagnosis and prognosis of patients with autoimmune myocarditis in the future	*FOXO3*<br>*Pknox1*	chrX	[19,91–94]
**miR-301**	• miR-301a and mir-302b are upregulated in DCM<br>• miR-301a promotes embryonic stem cell differentiation to cardiomyocytes and regulates Cofilin-2 which may impact DCM development	*Cfl2*<br>*PTEN*	hsa-mir-301a chr17<br>hsa-mir-301b chr22	[21,95,96]
**miR-302**	• miR-302a is downregulated in DCM, targets the cardiac-specific protein-leucine-rich repeat containing 10 (Lrrc10), which knockout in mice caused prenatal systolic dysfunction and was responsible for DCM development in postnatal life<br>• miR-302d promotes cardiomyocytes proliferation<br>• its inhibition may play an important role in protection against cardiomyocyte apoptosis during hypoxia/reoxygenation injury	*LATS2*<br>*Lrrc10*<br>*Mcl-1*	hsa-mir-302a chr4<br>hsa-mir-302b chr4<br>hsa-mir-302c chr4<br>hsa-mir-302d chr4<br>hsa-mir-302e chr11<br>hsa-mir-302f chr18	[47,97–99]
**miR-375**	• disrupts cardiomyocyte differentiation via influencing the Notch pathway<br>• upregulated in ToF, might be used as a prenatal marker of CHD in fetus	*Bcl2l2*<br>*FOXP1*<br>*LDHB*<br>*Notch2*	chr2	[26,100–102]

**miR-378**	• upregulated in ToF<br>• plays role in postnatal cardiac remodeling in mice<br>• it's secreted from cardiomyocytes after mechanical stress and acts as an inhibitor of excessive cardiac fibrosis through a paracrine mechanism<br>• attenuates ischemic injury in mice cardiomyocytes	*IGF1R*<br>*Eef1a2*<br>*Gns*<br>*Anks1*<br>*E2F2*<br>*MKK6*<br>*Purb*<br>*Mtf1*<br>*Nfx1*	hsa-mir-378a chr5<br>hsa-mir-378b chr3<br>hsa-mir-378c chr10<br>hsa-mir-378d-1 chr4<br>hsa-mir-378d-2 chr8<br>hsa-mir-378e chr5<br>hsa-mir-378f chr1<br>hsa-mir-378g unknown<br>hsa-mir-378h chr5<br>hsa-mir-378i chr22<br>hsa-mir-378j chr17	[12,103–106]
**miR-379**	• downregulated in VSD, targets genes related to cardiac development	*APOBEC2*<br>*ATF7*<br>*CCNB1*<br>*CDKN2AIP*<br>*CWF19L1*<br>*DENND2C*<br>*EIF4G2*<br>*EWSR1*<br>*GLIS1*<br>*GPBP1*<br>*IL28RA*<br>*KLHL14*<br>*LHX2*<br>*LMO4*<br>*MTM1*<br>*MSH6*<br>*NDN*<br>*NKX2-4*<br>*NR4A2*<br>*PAM*<br>*PCGF5*<br>*PDHX*<br>*PXT1*<br>*REPS1*<br>*SH3BGRL3*<br>*SLC20A1*<br>*SNX13*<br>*TOP2B*<br>*UBE2D2*<br>*UPK1B*	chr14	[18]

		UTP11L<br>*YARS*<br>*ZBTB26AKT1*<br>*ZDHHC20*		
**miR-381**	• downregulated in viral myocarditis, can bind both human and mouse cyclooxygenase (COX-2) mRNA to regulate their expression, may have a protective effect on endothelial cells against inflammation	*CXCR4*	chr14	[107–111]
**miR-409**	• downregulated in VSD		chr14	[18]
**miR-421**	• highly expressed in the RV tissue from infants with ToF, exists an inverse correlation between the expression of miR-421 and *SOX4*, a key regulator of the Notch and Wnt pathways	*SOX4*	chrX	[112]
**miR-424**	• targets heart development genes (*NF1* and *HAS2*), promotes cell proliferation and inhibits migration in primary embryonic mouse cardiomyocytes<br>• plays an important role in postischemic vascular remodeling and angiogenesis	*CUL2*<br>*HAS2*<br>*NF1*	chrX	[23,90]
**miR-433**	• downregulated in VSD, targets genes related to cardiac development	*ABCF2*<br>*ACAD8*<br>*ADRA1A*<br>*ATP5S*<br>*ATP6V0A1*<br>*B4GALT3*<br>*CDC27*<br>*CENPJ*<br>*CFTR*<br>*CHMP5*<br>*CLYBL*<br>*COX6B1*<br>*COX8A*<br>*COQ3*<br>*E2F3*<br>*EPHA5*<br>*FBXO5*<br>*GATA3*<br>*GEMIN5*<br>*GPATCH8*	chr14	[18]

		HAND1<br>*HIVEP1*<br>*HIVEP2*<br>*HP1BP3*<br>*HOXA5*<br>*IGFBP1*<br>*KIAA0195*<br>*LRRTM2*<br>*NMT2*<br>*NOTCH1*<br>*NME5*<br>*NR2F6*<br>*PAK4*<br>*PCCB*<br>*PPM1A*<br>*RAD9A*<br>*RECQL*<br>*SLC17A5*<br>*SMG5*<br>*SRFBP1*<br>*STMN4*<br>*SYNCRIP*<br>*TBC1D19*<br>*TDRD5*<br>*TIPRL*<br>*TRIP12*<br>*UBR1*<br>*WDR45L*<br>*ZC3H6*<br>*ZFPM2*<br>*XRCC6BP1*		
**miR-454**	• upregulated in DCM, and in patients with familial DCM caused by Lamin A/C (LMNA) gene mutations, targets insertion/deletion gene polymorphisms of angiotensin-converting enzyme, downregulated in diastolic dysfunction of the heart.	*ACE*	chr17	[13,47,71,113]
**miR-486**	• upregulated in ASD, VSD and AVSD and in parents of CHD children, especially mothers,	*Actn3*<br>*Atp5a1*<br>*Auh*<br>*Bhlhe40*	chr8	[17,114–116]

	• is modulated by stretch of cardiac muscle and increases left ventricle growth,<br>• may regulate cardiomyocyte apoptosis	*Bin1*<br>*Camk2a*<br>*Eif4g1*<br>*Lamp2*<br>*Mt2*<br>*Myh4*<br>*Myom1*<br>*M2*<br>*Naca*<br>*Pgam2*<br>*Phtf2*<br>*Pygm*<br>*Tnnc2*<br>*Trim63*<br>*Ttn*		
**miR-487**	• miR-487b is downregulated in VSD	*MAP2K4*<br>*NELF*<br>*ZNF219*	chr14	[18]
**miR-495**	• upregulated in DCM, exhibits sex-specific difference in expression, its inhibition in cardiomyocytes may attenuate the pathological hypertrophy	*NOD1*	chr14	[21]
**miR-498**	• upregulated in VSD, targets genes related to cardiac development	*ABTB1*<br>*ANKRD11*<br>*BECN1*<br>*CACNA1A*<br>*COL1A1*<br>*C9orf5*<br>*C10orf76*<br>*CRH*<br>*CRLS1*<br>*C5*<br>*DCBLD2*<br>*DEDD*<br>*KIAA1539*<br>*GALNT7*<br>*ITSN1*<br>*JHDM1D*<br>*JPH3*<br>*LPP*<br>*MAGEE1*	chr19	[18]

		MGA<br>*MTDH*<br>*NFIB*<br>*PAM*<br>*PCMTD1*<br>*RPRM*<br>*SACM1L*<br>*SLC25A12*<br>*SMAD4*<br>*SPAG16*<br>*ST8SIA2*<br>*TEX261*<br>*TIMM17A*<br>*TJP2*<br>*TNNT1*<br>*TRIM63*<br>*USP34*<br>*WDR47*<br>*ZEB2*		
**miR-499**	• participates in cardiac development, inhibits cardiomyocyte apoptosis<br>• upregulated in myocarditis<br>• upregulated in ToF<br>• increased in human and murine cardiac hypertrophy and cardiomyopathy	*Drp1*<br>*Dyrk2*<br>*KCNN3*<br>*Pacs2*<br>*Pdcd4*<br>*SOX6*<br>*ADAT2*<br>*AEBP2*<br>*ALPL*<br>*ANKRD13C*<br>*ANKRD40*<br>*ARHGAP23*<br>*ARHGAP32*<br>*ATF7*<br>*BTG1*<br>*CASK*<br>*COMMD2*<br>*COPS3*<br>*CPM*<br>*CPSF2*<br>*DCAF12*<br>*DCUN1D5*<br>*DENND4C*	chr20	[28,34,106,117–120]

		DNAJC15<br>*DNM1L*<br>*DPY19L3*<br>*EDNRB*<br>*EFNB1*<br>*ERLIN1*<br>*ERO1L*<br>*ETNK1*<br>*FAM168A*<br>*FAM169A*<br>*FGF2*<br>*FKBP5*<br>*FNDC3A*<br>*GMFB*<br>*H3F3B*<br>*ILF3*<br>*IRS2*<br>*JPH1*<br>*KATNAL1*<br>*KPNA3*<br>*LHFPL2*<br>*HMGIC*<br>*LRRC8A*<br>*MAMDC2*<br>*MARK2*<br>*MBNL3*<br>*MDM2*<br>*MED13L*<br>*MON2*<br>*MYLK3*<br>*NAP1L1*<br>*NIPA2*<br>*NPLOC4*<br>*NRIP1*<br>*NUFIP2*<br>*OSBPL1A*<br>*P4HA1*<br>*PDCD4*<br>*PECAM1*<br>*PGRMC2*<br>*PHF17*<br>*PPM1D*		

		PPP3CA<br>*PRKX*<br>*PTP4A1*<br>*RAB22A*<br>*RAB5C*<br>*RAB8B*<br>*RFFL*<br>*RNF114*<br>*ROD1*<br>*RRP15*<br>*RRP1B*<br>*SAMD4B*<br>*SAMD8*<br>*SEL1L*<br>*SF3B3*<br>*SH3BGRL2*<br>*SMAD4*<br>*SOS2*<br>*SPRED1*<br>*SPRY1*<br>*STK4*<br>*SUV39H1*<br>*SYAP1*<br>*SAP47*<br>*TBC1D15*<br>*TBC1*<br>*TCF12*<br>*TMBIM6*<br>*TMEM2*<br>*TMX1*<br>*TSPAN3*<br>*UBE2V1*<br>*USP47*<br>*WAC*<br>*WNK3*<br>*YAF2*<br>*YIPF6*<br>*ZDHHC2*<br>*ZDHHC9*		
**miR-518**	• miR-518f upregulated in DCM,<br>• plays role in improving myocardial ischemia	*Gzmb*<br>*ZBTB17*	chr19	[47,121,122]

**miR-543**	• upregulated viral myocarditis,<br>• may exhibit proangiogenic properties<br>• regulates myoblast proliferation and differentiation	*COL4A1*<br>*KLF6*<br>*SIRT1*	chr14	[87,123,124]
**miR-544**	• downregulated in DCM	*ANKRD1*	hsa-mir-544a chr14<br>hsa-mir-544b chr3	[47,125]
**miR-618**	• downregulated in DCM	*TPM1*	chr12	[47,126]
**miR-636**<br>**miR-639**<br>**miR-646**	• miR-636 is upregulated in pediatric patients with DCM<br>• miR-639 is downregulated in pediatric patients with DCM<br>• miR-646 is downregulated in pediatric patients with DCM		hsa-mir-636 chr17<br>hsa-mir-639 chr19<br>hsa-mir-646 chr20	[127]
**miR-875**	• downregulated in DCM	*MYPN*	chr8	[47,128]
**miR-1275**	• pregnancy related miRNA, may be used to distinguish fetuses with VSD<br>• by regulating the adrenergic signaling pathway may contribute to the progression of CHD<br>• influence cardiac muscle mitochondrial functioning<br>• play role in coronary heart disease	*PAPP-A*<br>*PRKACA*<br>*STK4*	chr6	[35,58,129]
**miR-3664**<br>**miR-4666**	• pregnancy related miRNAs, significantly different in VSD, both dysregulated in ToF<br>• miR-3664-3p showed significantly different expression in both SV and PTA	miR-3664 targets *PAPP-A* and *SAV1*	hsa-mir-3664 chr11<br>hsa-mir-4666a chr1<br>hsa-mir-4666b chrX	[58]

based on www.mirbase.org

Abbreviations: ASD- atrial septal defect, AVSD- atrioventricular septal defect, CHD- congenital heart disease, CVB3- Coxsackievirus B3, DCM- dilated cardiomyopathy, HLHS- hypoplastic left heart syndrome, FS- fractional shortening, LVEF- left ventricular ejection fraction, PAH- pulmonary arterial hypertension, PTA- persistent truncus arteriosus, SV- single ventricle, SVT – supraventricular tachycardia, ToF- Tetralofy of Fallot, VCM- viral cardiomyositis, VSD- ventricular septal defect

References

1. Sun, L.; Sun, S.; Zeng, S.; Li, Y.; Pan, W.; Zhang, Z. Expression of Circulating MicroRNA-1 and MicroRNA-133 in Pediatric Patients with Tachycardia. *Mol Med Rep* **2015**, *11*, 4039–4046, doi:10.3892/mmr.2015.3246.
2. Li, J.; Cao, Y.; Ma, X.J.; Wang, H.J.; Zhang, J.; Luo, X.; Chen, W.; Wu, Y.; Meng, Y.; Zhang, J.; et al. Roles of MiR-1-1 and MiR-181c in Ventricular Septal Defects. *Int J Cardiol* **2013**, *168*, 1441–1446, doi:10.1016/j.ijcard.2012.12.048.

3. Su, X.; Liang, H.; Wang, H.; Chen, G.; Jiang, H.; Wu, Q.; Liu, T.; Liu, Q.; Yu, T.; Gu, Y.; et al. Over-Expression of MicroRNA-1 Causes Arrhythmia by Disturbing Intracellular Trafficking System. *Sci Rep* **2017**, *7*, doi:10.1038/srep46259.
4. Physiology, C.; Luo, X.; Zhang, H.; Xiao, J.; Wang, Z. *Cellular Physiology Cellular Physiology Cellular Physiology Cellular Physiology Regulation of Human Cardiac Ion Channel Genes by MicroRNAs: Theoretical Perspective and Pathophysiological Implications *Authors with Equal Contribution*; 2010; Vol. 25;.
5. Zhang, Y.; Sun, L.; Zhang, Y.; Liang, H.; Li, X.; Cai, R.; Wang, L.; Du, W.; Zhang, R.; Li, J.; et al. Overexpression of MicroRNA-1 Causes Atrioventricular Block in Rodents. *Int J Biol Sci* **2013**, *9*, 445–462, doi:10.7150/ijbs.4630.
6. Wahl, C.M.; Schmidt, C.; Hecker, M.; Ullrich, N.D. Distress-Mediated Remodeling of Cardiac Connexin-43 in a Novel Cell Model for Arrhythmogenic Heart Diseases. *Int J Mol Sci* **2022**, *23*, doi:10.3390/ijms231710174.
7. Moric-Janiszewska, E.; Smolik, S.; Morka, A.; Szydłowski, L.; Kapral, M. Expression Levels of Serum Circulating MicroRNAs in Pediatric Patients with Ventricular and Supraventricular Arrhythmias. *Adv Med Sci* **2021**, *66*, 411–417, doi:10.1016/j.advms.2021.08.003.
8. Wang, D.; Li, T.; Cui, H.; Zhang, Y. Analysis of the Indicating Value of Cardiac Troponin I, Tumor Necrosis Factor-α, Interleukin-18, Mir-1 and Mir-146b for Viral Myocarditis among Children. *Cellular Physiology and Biochemistry* **2016**, *40*, 1325–1333, doi:10.1159/000453185.
9. Li, W.; Liu, M.; Zhao, C.; Chen, C.; Kong, Q.; Cai, Z.; Li, D. MiR-1/133 Attenuates Cardiomyocyte Apoptosis and Electrical Remodeling in Mice with Viral Myocarditis. *Cardiol J* **2020**, *27*, 285–294, doi:10.5603/CJ.a2019.0036.
10. Wang, B.; Shi, G.; Zhu, Z.; Chen, H.; Fu, Q. Sexual Difference of Small RNA Expression in Tetralogy of Fallot. *Sci Rep* **2018**, *8*, doi:10.1038/s41598-018-31243-6.
11. Sánchez-Gómez, M.C.; García-Mejía, K.A.; Pérez-Díaz Conti, M.; Díaz-Rosas, G.; Palma-Lara, I.; Sánchez-Urbina, R.; Klünder-Klünder, M.; Botello-Flores, J.A.; Balderrábano- Saucedo, N.A.; Contreras-Ramos, A. MicroRNAs Association in the Cardiac Hypertrophy Secondary to Complex Congenital Heart Disease in Children. *Pediatr Cardiol* **2017**, *38*, 991–1003, doi:10.1007/s00246-017-1607-8.
12. Grunert, M.; Appelt, S.; Dunkel, I.; Berger, F.; Sperling, S.R. Altered MicroRNA and Target Gene Expression Related to Tetralogy of Fallot. *Sci Rep* **2019**, *9*, doi:10.1038/s41598-019-55570-4.
13. Toro, R.; Blasco-Turrión, S.; Morales-Ponce, F.J.; Gonzalez, P.; Martínez-Camblor, P.; López-Granados, A.; Brugada, R.; Campuzano, O.; Pérez-Serra, A.; Rosa Longobardo, F.; et al. Plasma MicroRNAs as Biomarkers for Lamin A/C-Related Dilated Cardiomyopathy. *J Mol Med* **2018**, *96*, 845–856, doi:10.1007/s00109-018-1666-1.
14. Jiao, M.; You, H.Z.; Yang, X.Y.; Yuan, H.; Li, Y.L.; Liu, W.X.; Jin, M.; Du, J. Circulating MicroRNA Signature for the Diagnosis of Childhood Dilated Cardiomyopathy. *Sci Rep* **2018**, *8*, doi:10.1038/s41598-017-19138-4.
15. Satoh, M.; Minami, Y.; Takahashi, Y.; Tabuchi, T.; Nakamura, M. A Cellular MicroRNA, Let-7i, Is a Novel Biomarker for Clinical Outcome in Patients with Dilated Cardiomyopathy. *J Card Fail* **2011**, *17*, 923–929, doi:10.1016/j.cardfail.2011.07.012.
16. Zhou, X.; Sun, F.; Luo, S.; Zhao, W.; Yang, T.; Zhang, G.; Gao, M.; Lu, R.; Shu, Y.; Mu, W.; et al. Let-7a Is an Antihypertrophic Regulator in the Heart via Targeting Calmodulin. *Int J Biol Sci* **2017**, *13*, 22–31, doi:10.7150/ijbs.16298.
17. Song, Y.; Higgins, H.; Guo, J.; Harrison, K.; Schultz, E.N.; Hales, B.J.; Moses, E.K.; Goldblatt, J.; Pachter, N.; Zhang, G. Clinical Significance of Circulating MicroRNAs as Markers in Detecting and Predicting Congenital Heart Defects in Children. *J Transl Med* **2018**, *16*, doi:10.1186/s12967-018-1411-0.
18. Li, D.; Ji, L.; Liu, L.; Liu, Y.; Hou, H.; Yu, K.; Sun, Q.; Zhao, Z. Characterization of Circulating MicroRNA Expression in Patients with a Ventricular Septal Defect. *PLoS One* **2014**, *9*, doi:10.1371/journal.pone.0106318.

19. Gumus, G.; Giray, D.; Bobusoglu, O.; Tamer, L.; Karpuz, D.; Hallioglu, O. MicroRNA Values in Children with Rheumatic Carditis: A Preliminary Study. *Rheumatol Int* **2018**, *38*, 1199–1205, doi:10.1007/s00296-018-4069-2.
20. Toro, R.; Pérez-Serra, A.; Mangas, A.; Campuzano, O.; Sarquella-Brugada, G.; Quezada-Feijoo, M.; Ramos, M.; Alcalá, M.; Carrera, E.; García-Padilla, C.; et al. MiR-16-5p Suppression Protects Human Cardiomyocytes against Endoplasmic Reticulum and Oxidative Stress-Induced Injury. *Int J Mol Sci* **2022**, *23*, doi:10.3390/ijms23031036.
21. Hailu, F.T.; Karimpour-Fard, A.; Toni, L.S.; Bristow, M.R.; Miyamoto, S.D.; Stauffer, B.L.; Sucharov, C.C. Integrated Analysis of MiRNA–MRNA Interaction in Pediatric Dilated Cardiomyopathy. *Pediatr Res* **2022**, *92*, 98–108, doi:10.1038/s41390-021-01548-w.
22. Xu, X.; Su, Y. ling; Shi, J. yu; Lu, Q.; Chen, C. MicroRNA-17-5p Promotes Cardiac Hypertrophy by Targeting Mfn2 to Inhibit Autophagy. *Cardiovasc Toxicol* **2021**, *21*, 759–771, doi:10.1007/s12012-021-09667-w.
23. Staszel, T.; Zapała, B.; Polus, A.; Sadakierska-Chudy, A.; Kieć-Wilk, B.; Stępień, E.; Wybrańska, I.; Chojnacka, M.; Dembińska-Kieć, A. Role of MicroRNAs in Endothelial Cell Pathophysiology. *Pol Arch Med Wewn* **2011**, *121*, 361–366.
24. Huang, C.Y.; Pai, P.Y.; Kuo, C.H.; Ho, T.J.; Lin, J.Y.; Lin, D.Y.; Tsai, F.J.; Padma, V.V.; Kuo, W.W.; Huang, C.Y. P53-Mediated MiR-18 Repression Activates HSF2 for IGF-IIR-Dependent Myocyte Hypertrophy in Hypertension-Induced Heart Failure. *Cell Death Dis* **2017**, *8*, doi:10.1038/CDDIS.2017.320.
25. Chen, W.; Li, S. Circulating MicroRNA as a Novel Biomarker for Pulmonary Arterial Hypertension Due to Congenital Heart Disease. *Pediatr Cardiol* **2017**, *38*, 86–94, doi:10.1007/s00246-016-1487-3.
26. Zhu, S.; Cao, L.; Zhu, J.; Kong, L.; Jin, J.; Qian, L.; Zhu, C.; Hu, X.; Li, M.; Guo, X.; et al. Identification of Maternal Serum MicroRNAs as Novel Non-Invasive Biomarkers for Prenatal Detection of Fetal Congenital Heart Defects. *Clinica Chimica Acta* **2013**, *424*, 66–72, doi:10.1016/j.cca.2013.05.010.
27. Wang, H.; Bei, Y.; Shen, S.; Huang, P.; Shi, J.; Zhang, J.; Sun, Q.; Chen, Y.; Yang, Y.; Xu, T.; et al. MiR-21-3p Controls Sepsis-Associated Cardiac Dysfunction via Regulating SORBS2. *J Mol Cell Cardiol* **2016**, *94*, 43–53, doi:10.1016/j.yjmcc.2016.03.014.
28. Corsten, M.F.; Dennert, R.; Jochems, S.; Kuznetsova, T.; Devaux, Y.; Hofstra, L.; Wagner, D.R.; Staessen, J.A.; Heymans, S.; Schroen, B. Circulating MicroRNA-208b and MicroRNA-499 Reflect Myocardial Damage in Cardiovascular Disease. *Circ Cardiovasc Genet* **2010**, *3*, 499–506, doi:10.1161/CIRCGENETICS.110.957415.
29. Yang, L.; Wang, B.; Zhou, Q.; Wang, Y.; Liu, X.; Liu, Z.; Zhan, Z. MicroRNA-21 Prevents Excessive Inflammation and Cardiac Dysfunction after Myocardial Infarction through Targeting KBTBD7. *Cell Death Dis* **2018**, *9*, doi:10.1038/s41419-018-0805-5.
30. Gong, M.; Tao, L.; Li, X. MicroRNA-21-3p/Rcan1 Signaling Axis Affects Apoptosis of Cardiomyocytes of Sepsis Rats. *Gen Physiol Biophys* **2023**, *42*, 217–227, doi:10.4149/gpb_2022066.
31. Li, Y.; Sun, G.; Wang, L. MiR-21 Participates in LPS-Induced Myocardial Injury by Targeting Bcl-2 and CDK6. *Inflammation Research* **2022**, *71*, 205–214, doi:10.1007/s00011-021-01535-1.
32. Goldberg, L.; Tirosh-Wagner, T.; Vardi, A.; Abbas, H.; Pillar, N.; Shomron, N.; Nevo-Caspi, Y.; Paret, G. Circulating MicroRNAs: A Potential Biomarker for Cardiac Damage, Inflammatory Response, and Left Ventricular Function Recovery in Pediatric Viral Myocarditis. *J Cardiovasc Transl Res* **2018**, *11*, 319–328, doi:10.1007/s12265-018-9814-0.
33. Huang, Z.P.; Wang, D.Z. MiR-22 in Cardiac Remodeling and Disease. *Trends Cardiovasc Med* 2014, *24*, 267–272.
34. You, G.; Zu, B.; Wang, B.; Fu, Q.; Li, F. Identification of MiRNA–MRNA–TFs Regulatory Network and Crucial Pathways Involved in Tetralogy of Fallot. *Front Genet* **2020**, *11*, doi:10.3389/fgene.2020.00552.

35. Huang, G.-J.; Xie, X.-L.; Zou, Y. MiR-23b Targets GATA6 to down-Regulate IGF-1 and Promote the Development of Congenital Heart Disease. *Acta Cardiol* **2022**, *77*, 375–384, doi:10.1080/00015385.2021.1948207.
36. Jaguszewski, M.; Osipova, J.; Ghadri, J.R.; Napp, L.C.; Widera, C.; Franke, J.; Fijalkowski, M.; Nowak, R.; Fijalkowska, M.; Volkmann, I.; et al. A Signature of Circulating MicroRNAs Differentiates Takotsubo Cardiomyopathy from Acute Myocardial Infarction. *Eur Heart J* **2014**, *35*, 999–1006, doi:10.1093/eurheartj/eht392.
37. Icli, B.; Dorbala, P.; Feinberg, M.W. An Emerging Role for the MiR-26 Family in Cardiovascular Disease. *Trends Cardiovasc Med* 2014, *24*, 241–248.
38. Lozano-Velasco, E.; Galiano-Torres, J.; Jodar-Garcia, A.; Aranega, A.E.; Franco, D. MiR-27 and MiR-125 Distinctly Regulate Muscle-Enriched Transcription Factors in Cardiac and Skeletal Myocytes. *Biomed Res Int* **2015**, *2015*, doi:10.1155/2015/391306.
39. Yang, Q.; Wu, F.; Mi, Y.; Wang, F.; Cai, K.; Yang, X.; Zhang, R.; Liu, L.; Zhang, Y.; Wang, Y.; et al. Aberrant Expression of MiR-29b-3p Influences Heart Development and Cardiomyocyte Proliferation by Targeting NOTCH2. *Cell Prolif* **2020**, *53*, doi:10.1111/cpr.12764.
40. Wu, K.H.; Xiao, Q.R.; Yang, Y.; Xu, J.L.; Zhang, F.; Liu, C.M.; Zhang, Z.M.; Lu, Y.Q.; Huang, N.P. MicroRNA-34a Modulates the Notch Signaling Pathway in Mice with Congenital Heart Disease and Its Role in Heart Development. *J Mol Cell Cardiol* **2018**, *114*, 300–308, doi:10.1016/j.yjmcc.2017.11.015.
41. Bonauer, A.; Carmona, G.; Iwasaki, M.; Mione, M.; Koyanagi, M.; Fischer, A.; Burchfield, J.; Fox, H.; Doebele, C.; Ohtani, K.; et al. MicroRNA-92a Controls Angiogenesis and Functional Recovery of Ischemic Tissues in Mice. *Science (1979)* **2009**, *324*, 1710–1713, doi:10.1126/science.1174381.
42. Zhang, J.; Qin, L.; Han, L.; Zhao, Y.; Jing, H.; Song, W.; Shi, H. Role of MicroRNA-93 I in Pathogenesis of Left Ventricular Remodeling via Targeting Cyclin-D1. *Medical Science Monitor* **2017**, *23*, 3981–3988, doi:10.12659/MSM.897542.
43. Wo, Y.; Guo, J.; Li, P.; Yang, H.; Wo, J. Long Non-Coding RNA CHRF Facilitates Cardiac Hypertrophy through Regulating Akt3 via MiR-93. *Cardiovascular Pathology* **2018**, *35*, 29–36, doi:10.1016/j.carpath.2018.04.003.
44. Ramasamy, S.; Velmurugan, G.; Rekha, B.; Anusha, S.; Shanmugha Rajan, K.; Shanmugarajan, S.; Ramprasath, T.; Gopal, P.; Tomar, D.; Karthik, K. V.; et al. Egr-1 Mediated Cardiac MiR-99 Family Expression Diverges Physiological Hypertrophy from Pathological Hypertrophy. *Exp Cell Res* **2018**, *365*, 46–56, doi:10.1016/j.yexcr.2018.02.016.
45. Coppola, A.; Romito, A.; Borel, C.; Gehrig, C.; Gagnebin, M.; Falconnet, E.; Izzo, A.; Altucci, L.; Banfi, S.; Antonarakis, S.E.; et al. Cardiomyogenesis Is Controlled by the MiR-99a/Let-7c Cluster and Epigenetic Modifications. *Stem Cell Res* **2014**, *12*, 323–337, doi:10.1016/j.scr.2013.11.008.
46. Chen, D.; Chen, Z.; Jin, Y.; Dragas, D.; Zhang, L.; Adjei, B.S.; Wang, A.; Dai, Y.; Zhou, X. MicroRNA-99 Family Members Suppress Homeobox A1 Expression in Epithelial Cells. *PLoS One* **2013**, *8*, doi:10.1371/journal.pone.0080625.
47. Enes Coşkun, M.; Kervancioğlu, M.; Öztuzcu, S.; Yilmaz Coşkun, F.; Ergün, S.; Başpinar, O.; Kilinç, M.; Temel, L.; Coşkun, M.Y. Plasma MicroRNA Profiling of Children with Idiopathic Dilated Cardiomyopathy. *Biomarkers* **2016**, *21*, 56–61, doi:10.3109/1354750X.2015.1118533.
48. Sucharov, C.C.; Sucharov, J.; Karimpour-Fard, A.; Nunley, K.; Stauffer, B.L.; Miyamoto, S.D. Micro-RNA Expression in Hypoplastic Left Heart Syndrome. *J Card Fail* **2015**, *21*, 83–88, doi:10.1016/j.cardfail.2014.09.013.
49. Smolka, C.; Schlösser, D.; Koentges, C.; Tarkhnishvili, A.; Gorka, O.; Pfeifer, D.; Bemtgen, X.; Asmussen, A.; Groß, O.; Diehl, P.; et al. Cardiomyocyte-Specific MiR-100 Overexpression Preserves Heart Function under Pressure

Overload in Mice and Diminishes Fatty Acid Uptake as Well as ROS Production by Direct Suppression of Nox4 and CD36. *FASEB Journal* **2021**, *35*, doi:10.1096/fj.202100829RR.
50. Wong, L.L.; Wee, A.S.Y.; Lim, J.Y.; Ng, J.Y.X.; Chong, J.P.C.; Liew, O.W.; Lilyanna, S.; Martinez, E.C.; Ackers-Johnson, M.A.; Vardy, L.A.; et al. Natriuretic Peptide Receptor 3 (NPR3) Is Regulated by MicroRNA-100. *J Mol Cell Cardiol* **2015**, *82*, 13–21, doi:10.1016/j.yjmcc.2015.02.019.
51. Yuan, R.; Zhang, X.; Fang, Y.; Nie, Y.; Cai, S.; Chen, Y.; Mo, D. Mir-127-3p Inhibits the Proliferation of Myocytes by Targeting KMT5a. *Biochem Biophys Res Commun* **2018**, *503*, 970–976, doi:10.1016/j.bbrc.2018.06.104.
52. Li, J.; Wang, G.; Jiang, J.; Zhang, L.; Zhou, P.; Ren, H. MicroRNA-127-3p Regulates Myoblast Proliferation by Targeting Sept7. *Biotechnol Lett* **2020**, *42*, 1633–1644, doi:10.1007/s10529-020-02906-0.
53. Yaping, X.U.; Guotian, Y.I.N.; Dandan, J.I.A.; Jintao, D.O.U.; Xinyi, L.I.U.; Zhikun, G.U.O. Fibroblast-Derived Exosomal MiRNA-133 Promotes Cardiomyocyte-like Differentiation. *Acta Histochem* **2022**, *124*, 151931, doi:10.1016/J.ACTHIS.2022.151931.
54. Hedley, P.L.; Carlsen, A.L.; Christiansen, K.M.; Kanters, J.K.; Behr, E.R.; Corfield, V.A.; Christiansen, M. MicroRNAs in Cardiac Arrhythmia: DNA Sequence Variation of MiR-1 and MiR-133A in Long QT Syndrome. *Scand J Clin Lab Invest* **2014**, *74*, 485–491, doi:10.3109/00365513.2014.905696.
55. Zhang, Y.; Sun, L.; Sun, H.; Liu, X.; Luo, X.; Li, C.; Sun, D.; Li, T. Overexpression of MicroRNA-133b Reduces Myocardial Injuries in Children with Viral Myocarditis by Targeting Rab27B Gene. *Cell Mol Biol* **2017**, *6*, doi:10.14715/cmb/2017.63.10.13.
56. Sharma, S.; Liu, J.; Wei, J.; Yuan, H.; Zhang, T.; Bishopric, N.H. Repression of MiR-142 by P300 and MAPK Is Required for Survival Signalling via Gp130 during Adaptive Hypertrophy. *EMBO Mol Med* **2012**, *4*, 617–632, doi:10.1002/emmm.201200234.
57. Liu, B. lei; Cheng, M.; Hu, S.; Wang, S.; Wang, L.; Tu, X.; Huang, C. xin; Jiang, H.; Wu, G. Overexpression of MiR-142-3p Improves Mitochondrial Function in Cardiac Hypertrophy. *Biomedicine and Pharmacotherapy* **2018**, *108*, 1347–1356, doi:10.1016/j.biopha.2018.09.146.
58. Gu, H.; Chen, L.; Xue, J.; Huang, T.; Wei, X.; Liu, D.; Ma, W.; Cao, S.; Yuan, Z. Expression Profile of Maternal Circulating MicroRNAs as Non-Invasive Biomarkers for Prenatal Diagnosis of Congenital Heart Defects. *Biomedicine and Pharmacotherapy* **2019**, *109*, 823–830, doi:10.1016/j.biopha.2018.10.110.
59. Yu, B.; Zhao, Y.; Zhang, H.; Xie, D.; Nie, W.; Shi, K. Inhibition of MicroRNA-143-3p Attenuates Myocardial Hypertrophy by Inhibiting Inflammatory Response. *Cell Biol Int* **2018**, *42*, 1584–1593, doi:10.1002/cbin.11053.
60. Ogawa, K.; Noda, A.; Ueda, J.; Ogata, T.; Matsuyama, R.; Nishizawa, Y.; Qiao, S.; Iwata, S.; Ito, M.; Fujihara, Y.; et al. Forced Expression of MiR-143 and -145 in Cardiomyocytes Induces Cardiomyopathy with a Reductive Redox Shift. *Cell Mol Biol Lett* **2020**, *25*, doi:10.1186/s11658-020-00232-x.
61. Deacon, D.C.; Nevis, K.R.; Cashman, T.J.; Zhou, Y.; Zhao, L.; Washko, D.; Guner-Ataman, B.; Burns, C.G.; Burns, C.E. The MiR-143-Adducin3 Pathway Is Essential for Cardiac Chamber Morphogenesis. *Development* **2010**, *137*, 1887–1896, doi:10.1242/dev.050526.
62. Wang, L.; Tian, D.; Hu, J.; Xing, H.; Sun, M.; Wang, J.; Jian, Q.; Yang, H. MiRNA-145 Regulates the Development of Congenital Heart Disease Through Targeting FXN. *Pediatr Cardiol* **2016**, *37*, 629–636, doi:10.1007/s00246-015-1325-z.
63. Di, Y.-F.; Li, D.-C.; Shen, Y.-Q.; Wang, C.-L.; Zhang, D.-Y.; Shang, A.-Q.; Hu, T. *MiR-146b Protects Cardiomyocytes Injury in Myocardial Ischemia/Reperfusion by Targeting Smad4*; 2017; Vol. 9;.
64. Chouvarine, P.; Legchenko, E.; Geldner, J.; Riehle, C.; Hansmann, G. Hypoxia Drives Cardiac MiRNAs and Inflammation in the Right and Left Ventricle. *J Mol Med* **2019**, *97*, 1427–1438, doi:10.1007/s00109-019-01817-6.

65. Cheng, H.S.; Sivachandran, N.; Lau, A.; Boudreau, E.; Zhao, J.L.; Baltimore, D.; Delgado-Olguin, P.; Cybulsky, M.I.; Fish, J.E. MicroRNA-146 Represses Endothelial Activation by Inhibiting pro-Inflammatory Pathways. *EMBO Mol Med* **2013**, *5*, 1017–1034, doi:10.1002/emmm.201202318.
66. Liu, Y.L.; Wu, W.F.; Xue, Y.; Gao, M.; Yan, Y.; Kong, Q.; Pang, Y.; Yang, F. MicroRNA-21 and -146b Are Involved in the Pathogenesis of Murine Viral Myocarditis by Regulating TH-17 Differentiation. *Arch Virol* **2013**, *158*, 1953–1963, doi:10.1007/s00705-013-1695-6.
67. Jin, Y.; Ai, L.; Chai, X.; Tang, P.; Zhang, W.; Yang, L.; Hu, Y.; Xu, Y.; Li, S. Maternal Circulating Exosomal MiRNAs as Non-Invasive Biomarkers for the Prediction of Fetal Ventricular Septal Defect. *Front Genet* **2021**, *12*, doi:10.3389/fgene.2021.717208.
68. Zhang, Y.; Peng, B.; Han, Y. MiR-182 Alleviates the Development of Cyanotic Congenital Heart Disease by Suppressing HES1. *Eur J Pharmacol* **2018**, *836*, 18–24, doi:10.1016/j.ejphar.2018.08.013.
69. Huang, J.; Li, X.; Li, H.; Su, Z.; Wang, J.; Zhang, H. *Down-Regulation of MicroRNA-184 Contributes to the Development of Cyanotic Congenital Heart Diseases*; 2015; Vol. 8;.
70. Ektesabi, A.M.; Mori, K.; Tsoporis, J.; Walsh, C.; Mai, S.; Hu, P.; DosSantos, C. REGULATION OF MIR-187B IN ENDOTOXEMIC PRIMARY CARDIOMYOCYTES AND SEPTIC MURINE HEARTS TREATED WITH MESENCHYMAL STROMAL/STEM CELLS. *Canadian Journal of Cardiology* **2019**, *35*, S48, doi:10.1016/j.cjca.2019.07.444.
71. Fayez, A.G.; Esmaiel, N.N.; Salem, S.M.; Ashaat, E.A.; El-Saiedi, S.A.; El Ruby, M.O. MiR-454-3p and MiR-194-5p Targeting Cardiac Sarcolemma Ion Exchange Transcripts Are Potential Noninvasive Diagnostic Biomarkers for Childhood Dilated Cardiomyopathy in Egyptian Patients. *Egyptian Heart Journal* **2022**, *74*, doi:10.1186/s43044-022-00300-x.
72. Friedrichs, F.; Zugck, C.; Rauch, G.J.; Ivandic, B.; Weichenhan, D.; Müller-Bardorff, M.; Meder, B.; Mokhtari, N.E. El; Regitz-Zagrosek, V.; Hetzer, R.; et al. HBEGF, SRA1, and IK: Three Cosegregating Genes as Determinants of Cardiomyopathy. *Genome Res* **2009**, *19*, 395–403, doi:10.1101/gr.076653.108.
73. Wang, L.; Qin, D.; Shi, H.; Zhang, Y.; Li, H.; Han, Q. MiR-195-5p Promotes Cardiomyocyte Hypertrophy by Targeting MFN2 and FBXW7. *Biomed Res Int* **2019**, *2019*, doi:10.1155/2019/1580982.
74. Zhou, Y.; Jia, W.K.; Jian, Z.; Zhao, L.; Liu, C.C.; Wang, Y.; Xiao, Y. Bin Downregulation of MicroRNA-199a-5p Protects Cardiomyocytes in Cyanotic Congenital Heart Disease by Attenuating Endoplasmic Reticulum Stress. *Mol Med Rep* **2017**, *16*, 2992–3000, doi:10.3892/mmr.2017.6934.
75. Li, X.; Xiang, D.; Shu, Y.; Hu, K.; Zhang, Y.; Li, Y. MicroRNA-204 as an Indicator of Severity of Pulmonary Hypertension in Children with Congenital Heart Disease Complicated with Pulmonary Hypertension. *Medical Science Monitor* **2019**, *25*, 10173–10179, doi:10.12659/MSM.917662.
76. Cheng, X.; Du, J.; Shen, L.; Tan, Z.; Jiang, D.; Jiang, A.; Li, Q.; Tang, G.; Jiang, Y.; Wang, J.; et al. MiR-204-5p Regulates C2C12 Myoblast Differentiation by Targeting MEF2C and ERRγ. *Biomedicine and Pharmacotherapy* **2018**, *101*, 528–535, doi:10.1016/j.biopha.2018.02.096.
77. Seeger, T.S.; Frank, D.; Rohr, C.; Will, R.; Just, S.; Grund, C.; Lyon, R.; Luedde, M.; Koegl, M.; Sheikh, F.; et al. Myozap, a Novel Intercalated Disc Protein, Activates Serum Response Factor-Dependent Signaling and Is Required to Maintain Cardiac Function in Vivo. *Circ Res* **2010**, *106*, 880–890, doi:10.1161/CIRCRESAHA.109.213256.
78. Wang, T.; Li, T.; Niu, X.; Hu, L.; Cheng, J.; Guo, D.; Ren, H.; Zhao, R.; Ji, Z.; Liu, P.; et al. ADSC-Derived Exosomes Attenuate Myocardial Infarction Injury by Promoting MiR-205-Mediated Cardiac Angiogenesis. *Biol Direct* **2023**, *18*, doi:10.1186/s13062-023-00361-1.

79. Xuan, Y.; Liu, S.; Li, Y.; Dong, J.; Luo, J.; Liu, T.; Jin, Y.; Sun, Z. Short-Term Vagus Nerve Stimulation Reduces Myocardial Apoptosis by Downregulating MicroRNA-205 in Rats with Chronic Heart Failure. *Mol Med Rep* **2017**, *16*, 5847–5854, doi:10.3892/mmr.2017.7344.
80. Salant, G.M.; Tat, K.L.; Goodrich, J.A.; Kugel, J.F. MiR-206 Knockout Shows It Is Critical for Myogenesis and Directly Regulates Newly Identified Target MRNAs. *RNA Biol* **2020**, *17*, 956–965, doi:10.1080/15476286.2020.1737443.
81. Anderson, C.; Catoe, H.; Werner, R. MIR-206 Regulates Connexin43 Expression during Skeletal Muscle Development. *Nucleic Acids Res* **2006**, *34*, 5863–5871, doi:10.1093/nar/gkl743.
82. Devaux, Y.; Vausort, M.; Goretti, E.; Nazarov, P. V.; Azuaje, F.; Gilson, G.; Corsten, M.F.; Schroen, B.; Lair, M.L.; Heymans, S.; et al. Use of Circulating MicroRNAs to Diagnose Acute Myocardial Infarction. *Clin Chem* **2012**, *58*, 559–567, doi:10.1373/clinchem.2011.173823.
83. Callis, T.E.; Pandya, K.; Hee, Y.S.; Tang, R.H.; Tatsuguchi, M.; Huang, Z.P.; Chen, J.F.; Deng, Z.; Gunn, B.; Shumate, J.; et al. MicroRNA-208a Is a Regulator of Cardiac Hypertrophy and Conduction in Mice. *Journal of Clinical Investigation* **2009**, *119*, 2772–2786, doi:10.1172/JCI36154.
84. Huang, X.-H.; Li, J.-L.; Li, X.-Y.; Wang, S.-X.; Jiao, Z.-H.; Li, S.-Q.; Liu, J.; Ding, J. MiR-208a in Cardiac Hypertrophy and Remodeling. *Front Cardiovasc Med* **2021**, *8*, doi:10.3389/fcvm.2021.773314.
85. Zloto, K.; Tirosh-Wagner, T.; Bolkier, Y.; Bar-Yosef, O.; Vardi, A.; Mishali, D.; Paret, G.; Nevo-Caspi, Y. Preoperative MiRNA-208a as a Predictor of Postoperative Complications in Children with Congenital Heart Disease Undergoing Heart Surgery. *J Cardiovasc Transl Res* **2020**, *13*, 245–252, doi:10.1007/s12265-019-09921-1.
86. Zhao, X.; Wang, Y.; Sun, X. The Functions of MicroRNA-208 in the Heart. *Diabetes Res Clin Pract* 2020, *160*.
87. Xia, K.; Zhang, Y.; Sun, D. MiR-217 and MiR-543 Downregulation Mitigates Inflammatory Response and Myocardial Injury in Children with Viral Myocarditis by Regulating the SIRT1/AMPK/NF-κB Signaling Pathway. *Int J Mol Med* **2020**, *45*, 634–646, doi:10.3892/ijmm.2019.4442.
88. Liu, J.J.; Zhao, C.M.; Li, Z.G.; Wang, Y.M.; Miao, W.; Wu, X.J.; Wang, W.J.; Liu, C.; Wang, D.; Wang, K.; et al. MiR-218 Involvement in Cardiomyocyte Hypertrophy Is Likely through Targeting REST. *Int J Mol Sci* **2016**, *17*, doi:10.3390/ijms17060848.
89. Hu, C.; Huang, S.; Wu, F.; Ding, H. MicroRNA-219-5p Participates in Cyanotic Congenital Heart Disease Progression by Regulating Cardiomyocyte Apoptosis. *Exp Ther Med* **2020**, *21*, 1–1, doi:10.3892/etm.2020.9468.
90. Zhang, J.; Chang, J.J.; Xu, F.; Ma, X.J.; Wu, Y.; Li, W.C.; Wang, H.J.; Huang, G.Y.; Ma, D. MicroRNA Deregulation in Right Ventricular Outflow Tract Myocardium in Nonsyndromic Tetralogy of Fallot. *Canadian Journal of Cardiology* **2013**, *29*, 1695–1703, doi:10.1016/j.cjca.2013.07.002.
91. Pan, L.; Yan, B.; Zhang, J.; Zhao, P.; Jing, Y.; Yu, J.; Hui, J.; Lu, Q. Mesenchymal Stem Cells-Derived Extracellular Vesicles-Shuttled MicroRNA-223-3p Suppress Lipopolysaccharide-Induced Cardiac Inflammation, Pyroptosis, and Dysfunction. *Int Immunopharmacol* **2022**, *110*, 108910, doi:10.1016/J.INTIMP.2022.108910.
92. Stauffer, B.L.; Russell, G.; Nunley, K.; Miyamoto, S.D.; Sucharov, C.C. MiRNA Expression in Pediatric Failing Human Heart. *J Mol Cell Cardiol* **2013**, *57*, 43–46, doi:10.1016/j.yjmcc.2013.01.005.
93. Chen, L.; Hou, X.; Zhang, M.; Zheng, Y.; Zheng, X.; Yang, Q.; Li, J.; Gu, N.; Zhang, M.; Sun, Y.; et al. MicroRNA-223-3p Modulates Dendritic Cell Function and Ameliorates Experimental Autoimmune Myocarditis by Targeting the NLRP3 Inflammasome. *Mol Immunol* **2020**, *117*, 73–83, doi:10.1016/j.molimm.2019.10.027.
94. Gou, W.; Zhang, Z.; Yang, C.; Li, Y. MiR-223/Pknox1 Axis Protects Mice from CVB3-Induced Viral Myocarditis by Modulating Macrophage Polarization. *Exp Cell Res* **2018**, *366*, 41–48, doi:10.1016/j.yexcr.2018.03.004.

95. Rangrez, A.Y.; Hoppe, P.; Kuhn, C.; Zille, E.; Frank, J.; Frey, N.; Frank, D. MicroRNA MiR-301a Is a Novel Cardiac Regulator of Cofilin-2. *PLoS One* **2017**, *12*, doi:10.1371/journal.pone.0183901.

96. Zhen, L.-X.; Gu, Y.-Y.; Zhao, Q.; Zhu, H.-F.; Lü, J.-H.; Li, S.-J.; Xu, Z.; Li, L.; Yu, Z.-R. MiR-301a Promotes Embryonic Stem Cell Differentiation to Cardiomyocytes. *World J Stem Cells* **2019**, *11*, 1130–1141, doi:10.4252/wjsc.v11.i12.1130.

97. Xu, F.; Yang, J.; Shang, J.; Lan, F.; Li, M.; Shi, L.; Shen, L.; Wang, Y.; Ge, J. MicroRNA-302d Promotes the Proliferation of Human Pluripotent Stem Cell-Derived Cardiomyocytes by Inhibiting LATS2 in the Hippo Pathway. *Clin Sci* **2019**, *133*, 1387–1399, doi:10.1042/CS20190099.

98. Fang, Y.C.; Yeh, C.H. Inhibition of MIR-302 Suppresses Hypoxia-Reoxygenation-Induced H9c2 Cardiomyocyte Death by Regulating Mcl-1 Expression. *Oxid Med Cell Longev* **2017**, *2017*, doi:10.1155/2017/7968905.

99. Brody, M.J.; Hacker, T.A.; Patel, J.R.; Feng, L.; Sadoshima, J.; Tevosian, S.G.; Balijepalli, R.C.; Moss, R.L.; Lee, Y. Ablation of the Cardiac-Specific Gene Leucine-Rich Repeat Containing 10 (Lrrc10) Results in Dilated Cardiomyopathy. *PLoS One* **2012**, *7*, doi:10.1371/journal.pone.0051621.

100. Wang, L.; Song, G.; Liu, M.; Chen, B.; Chen, Y.; Shen, Y.; Zhu, J.; Zhou, X. MicroRNA-375 Overexpression Influences P19 Cell Proliferation, Apoptosis and Differentiation through the Notch Signaling Pathway. *Int J Mol Med* **2016**, *37*, 47–55, doi:10.3892/ijmm.2015.2399.

101. Li, Y.; Li, X.; Wang, L.; Han, N.; Yin, G. MiR-375-3p Contributes to Hypoxia-Induced Apoptosis by Targeting Forkhead Box P1 (FOXP1) and Bcl2 like Protein 2 (Bcl2l2) in Rat Cardiomyocyte H9c2 Cells. *Biotechnol Lett* **2021**, *43*, 353–367, doi:10.1007/s10529-020-03013-w.

102. Feng, H.; Wu, J.; Chen, P.; Wang, J.; Deng, Y.; Zhu, G.; Xian, J.; Huang, L.; Ouyang, W. MicroRNA-375-3p Inhibitor Suppresses Angiotensin II-Induced Cardiomyocyte Hypertrophy by Promoting Lactate Dehydrogenase B Expression. *J Cell Physiol* **2019**, *234*, 14198–14209, doi:10.1002/jcp.28116.

103. Knezevic, I.; Patel, A.; Sundaresan, N.R.; Gupta, M.P.; Solaro, R.J.; Nagalingam, R.S.; Gupta, M. A Novel Cardiomyocyte-Enriched MicroRNA, MiR-378, Targets Insulin-like Growth Factor 1 Receptor: Implications in Postnatal Cardiac Remodeling and Cell Survival. *Journal of Biological Chemistry* **2012**, *287*, 12913–12926, doi:10.1074/jbc.M111.331751.

104. Yuan, J.; Liu, H.; Gao, W.; Zhang, L.; Ye, Y.; Yuan, L.; Ding, Z.; Wu, J.; Kang, L.; Zhang, X.; et al. MicroRNA-378 Suppresses Myocardial Fibrosis through a Paracrine Mechanism at the Early Stage of Cardiac Hypertrophy Following Mechanical Stress. *Theranostics* **2018**, *8*, 2565–2582, doi:10.7150/thno.22878.

105. Fang, J.; Song, X.W.; Tian, J.; Chen, H.Y.; Li, D.F.; Wang, J.F.; Ren, A.J.; Yuan, W.J.; Lin, L. Overexpression of MicroRNA-378 Attenuates Ischemia-Induced Apoptosis by Inhibiting Caspase-3 Expression in Cardiac Myocytes. *Apoptosis* **2012**, *17*, 410–423, doi:10.1007/s10495-011-0683-0.

106. Matkovich, S.J.; Hu, Y.; Dorn, G.W. Regulation of Cardiac MicroRNAs by Cardiac MicroRNAs. *Circ Res* **2013**, *113*, 62–71, doi:10.1161/CIRCRESAHA.113.300975.

107. Liu, J.; Yang, Y.; Lu, R.; Liu, Q.; Hong, S.; Zhang, Z.; Hu, G. MicroRNA-381-3p Signatures as a Diagnostic Marker in Patients with Sepsis and Modulates Sepsis-Steered Cardiac Damage and Inflammation by Binding HMGB1. *Bioengineered* **2021**, *12*, 11936–11946, doi:10.1080/21655979.2021.2006967.

108. Lu, L.; Zhang, H.; Dong, W.; Peng, W.; Yang, J. MiR-381 Negatively Regulates Cardiomyocyte Survival by Suppressing Notch Signaling. *In Vitro Cell Dev Biol Anim* **2018**, *54*, 610–619, doi:10.1007/s11626-018-0277-z.

109. Zhang, Y.; Sun, L.; Sun, H.; Yu, Z.; Liu, X.; Luo, X.; Li, C.; Sun, D.; Li, T. MicroRNA-381 Protects Myocardial Cell Function in Children and Mice with Viral Myocarditis via Targeting Cyclooxygenase-2 Expression. *Exp Ther Med* **2018**, *15*, 5510–5516, doi:10.3892/etm.2018.6082.

110. Li, Y.; Huang, J.; Yan, H.; Li, X.; Ding, C.; Wang, Q.; Lu, Z. Protective Effect of MicroRNA-381 against Inflammatory Damage of Endothelial Cells during Coronary Heart Disease by Targeting CXCR4. *Mol Med Rep* **2020**, *21*, 1439–1448, doi:10.3892/mmr.2020.10957.
111. Li, Y.; Huang, J.; Yan, H.; Li, X.; Ding, C.; Wang, Q.; Lu, Z. Protective Effect of MicroRNA-381 against Inflammatory Damage of Endothelial Cells during Coronary Heart Disease by Targeting CXCR4. *Mol Med Rep* **2020**, *21*, 1439–1448, doi:10.3892/mmr.2020.10957.
112. Bittel, D.C.; Kibiryeva, N.; Marshall, J.A.; O'Brien, J.E. MicroRNA-421 Dysregulation Is Associated with Tetralogy of Fallot. *Cells* **2014**, *3*, 713–723, doi:10.3390/cells3030713.
113. Nair, N.; Kumar, S.; Gongora, E.; Gupta, S. Circulating MiRNA as Novel Markers for Diastolic Dysfunction. *Mol Cell Biochem* **2013**, *376*, 33–40, doi:10.1007/s11010-012-1546-x.
114. Samani, A.; Hightower, R.M.; Reid, A.L.; English, K.G.; Lopez, M.A.; Scott Doyle, J.; Conklin, M.J.; Schneider, D.A.; Bamman, M.M.; Widrick, J.J.; et al. MiR-486 Is Essential for Muscle Function and Suppresses a Dystrophic Transcriptome. *Life Sci Alliance* **2022**, *5*, doi:10.26508/lsa.202101215.
115. Lange, S.; Banerjee, I.; Carrion, K.; Serrano, R.; Habich, L.; Kameny, R.; Lengenfelder, L.; Dalton, N.; Meili, R.; Börgeson, E.; et al. MiR-486 Is Modulated by Stretch and Increases Ventricular Growth. *JCI Insight* **2019**, *4*, 1–17, doi:10.1172/jci.insight.125507.
116. Sun, Y.; Su, Q.; Li, L.; Wang, X.; Lu, Y.; Liang, J. MiR-486 Regulates Cardiomyocyte Apoptosis by P53-Mediated BCL-2 Associated Mitochondrial Apoptotic Pathway. *BMC Cardiovasc Disord* **2017**, *17*, doi:10.1186/s12872-017-0549-7.
117. Ling, T.Y.; Wang, X.L.; Chai, Q.; Lau, T.W.; Koestler, C.M.; Park, S.J.; Daly, R.C.; Greason, K.L.; Jen, J.; Wu, L.Q.; et al. Regulation of the SK3 Channel by MicroRNA-499 - Potential Role in Atrial Fibrillation. *Heart Rhythm* **2013**, *10*, 1001–1009, doi:10.1016/j.hrthm.2013.03.005.
118. Wang, J.; Jia, Z.; Zhang, C.; Sun, M.; Wang, W.; Chen, P.; Ma, K.; Zhang, Y.; Li, X.; Zhou, C. MiR-499 Protects Cardiomyocytes from H2O2-Induced Apoptosis via Its Effects on Pdcd4 and Pacs2. *RNA Biol* **2014**, *11*, 339–350, doi:10.4161/rna.28300.
119. Shi, Y.; Han, Y.; Niu, L.; Li, J.; Chen, Y. MiR-499 Inhibited Hypoxia/Reoxygenation Induced Cardiomyocytes Injury by Targeting SOX6. *Biotechnol Lett* **2019**, *41*, 837–847, doi:10.1007/s10529-019-02685-3.
120. Matkovich, S.J.; Hu, Y.; Eschenbacher, W.H.; Dorn, L.E.; Dorn, G.W. Direct and Indirect Involvement of MicroRNA-499 in Clinical and Experimental Cardiomyopathy. *Circ Res* **2012**, *111*, 521–531, doi:10.1161/CIRCRESAHA.112.265736.
121. Li, X.; Luo, R.; Mo, X.; Jiang, R.; Kong, H.; Hua, W.; Wu, X. Polymorphism of ZBTB17 Gene Is Associated with Idiopathic Dilated Cardiomyopathy: A Case Control Study in a Han Chinese Population. *Eur J Med Res* **2013**, *18*, doi:10.1186/2047-783X-18-10.
122. Yang, H.; Su, J.; Meng, W.; Chen, X.; Xu, Y.; Sun, B. Mir-518a-5p Targets Gzmb to Extenuate Vascular Endothelial Cell Injury Induced by Hypoxia-Reoxygenation and Thereby Improves Myocardial Ischemia. *Int Heart J* **2021**, *62*, 658–665, doi:10.1536/ihj.20-619.
123. Kang, T.; Xing, W.; Xi, Y.; Chen, K.; Zhan, M.; Tang, X.; Wang, Y.; Zhang, R.; Lei, M. MiR-543 Regulates Myoblast Proliferation and Differentiation of C2C12 Cells by Targeting KLF6. *J Cell Biochem* **2020**, *121*, 4827–4837, doi:10.1002/jcb.29710.
124. Yang, M.; Liu, X.; Jiang, M.; Li, J.; Tang, Y.; Zhou, L. MiR-543 in Human Mesenchymal Stem Cell–Derived Exosomes Promotes Cardiac Microvascular Endothelial Cell Angiogenesis after Myocardial Infarction through COL4A1. *IUBMB Life* **2021**, *73*, 927–940, doi:10.1002/iub.2474.

125. Duboscq-Bidot, L.; Charron, P.; Ruppert, V.; Fauchier, L.; Richter, A.; Tavazzi, L.; Arbustini, E.; Wichter, T.; Maisch, B.; Komajda, M.; et al. Mutations in the ANKRD1 Gene Encoding CARP Are Responsible for Human Dilated Cardiomyopathy. *Eur Heart J* **2009**, *30*, 2128–2136, doi:10.1093/eurheartj/ehp225.
126. Redwood, C.; Robinson, P. Alpha-Tropomyosin Mutations in Inherited Cardiomyopathies. *J Muscle Res Cell Motil* 2013, *34*, 285–294.
127. Miyamoto, S.D.; Karimpour-Fard, A.; Peterson, V.; Auerbach, S.R.; Stenmark, K.R.; Stauffer, B.L.; Sucharov, C.C. Circulating MicroRNA as a Biomarker for Recovery in Pediatric Dilated Cardiomyopathy. *Journal of Heart and Lung Transplantation* **2015**, *34*, 724–733, doi:10.1016/j.healun.2015.01.979.
128. Meyer, T.; Ruppert, V.; Ackermann, S.; Richter, A.; Perrot, A.; Sperling, S.R.; Posch, M.G.; Maisch, B.; Pankuweit, S. Novel Mutations in the Sarcomeric Protein Myopalladin in Patients with Dilated Cardiomyopathy. *European Journal of Human Genetics* **2013**, *21*, 294–300, doi:10.1038/ejhg.2012.173.
129. Nymark, P.; Wijshoff, P.; Cavill, R.; Van Herwijnen, M.; Coonen, M.L.J.; Claessen, S.; Catalán, J.; Norppa, H.; Kleinjans, J.C.S.; Briedé, J.J. Extensive Temporal Transcriptome and MicroRNA Analyses Identify Molecular Mechanisms Underlying Mitochondrial Dysfunction Induced by Multi-Walled Carbon Nanotubes in Human Lung Cells. *Nanotoxicology* **2015**, *9*, 624–635, doi:10.3109/17435390.2015.1017022.
